# Tonsillar Microbiome‐Derived Lantibiotics Induce Structural Changes of IL‐6 and IL‐21 Receptors and Modulate Host Immunity

**DOI:** 10.1002/advs.202202706

**Published:** 2022-08-28

**Authors:** Jing Li, Jiayang Jin, Shenghui Li, Yan Zhong, Yuebo Jin, Xuan Zhang, Binbin Xia, Yinhua Zhu, Ruochun Guo, Xiaolin Sun, Jianping Guo, Fanlei Hu, Wenjing Xiao, Fei Huang, Hua Ye, Ru Li, Yunshan Zhou, Xiaohong Xiang, Haihong Yao, Qiulong Yan, Li Su, Lijun Wu, Tuoping Luo, Yudong Liu, Xiaohuan Guo, Junjie Qin, Hai Qi, Jing He, Jun Wang, Zhanguo Li

**Affiliations:** ^1^ Department of Rheumatology and Immunology Peking University People's Hospital Beijing 100044 China; ^2^ Beijing Key Laboratory for Rheumatism Mechanism and Immune Diagnosis (BZ0135) Beijing 100044 China; ^3^ Key Laboratory of Precision Nutrition and Food Quality Department of Nutrition and Health China Agricultural University Beijing 100083 China; ^4^ Promegene Translational Research Institute Shenzhen 518110 China; ^5^ Department of Rheumatology and Immunology People's Hospital of Xin Jiang Uygur Autonomous Region Urumqi 830001 China; ^6^ CAS Key Laboratory for Pathogenic Microbiology and Immunology Institute of Microbiology Chinese Academy of Sciences Beijing 100101 China; ^7^ University of Chinese Academy of Sciences Beijing 100049 China; ^8^ Peking‐Tsinghua Center for Life Sciences Academy for Advanced Interdisciplinary Studies Peking University Beijing 100871 China; ^9^ Key Laboratory of Bioorganic Chemistry and Molecular Engineering Ministry of Education and Beijing National Laboratory for Molecular Science College of Chemistry and Molecular Engineering Peking University Beijing 100871 China; ^10^ Emergency Department Peking University People's Hospital Beijing 100044 China; ^11^ Department of Microbiology College of Basic Medical Sciences Dalian Medical University Dalian 116044 China; ^12^ Center of Medical and Health Analysis Peking University Beijing 100191 China; ^13^ Department of Clinical Laboratory Peking University People's Hospital Beijing 100044 China; ^14^ Institute for Immunology, School of Medicine Tsinghua University Beijing 100084 China; ^15^ Department of Basic Biomedical Sciences, School of Medicine, Laboratory of Dynamic Immunobiology, Tsinghua‐Peking Center for Life Sciences Tsinghua University Beijing 10084 China; ^16^ State Key Laboratory of Natural and Biomimetic Drugs School of Pharmaceutical Sciences Peking University Beijing 100191 China

**Keywords:** IL‐6 and IL‐21 receptor, lantibiotics, tonsillar microbiome, rheumatoid arthritis, salivaricins

## Abstract

Emerging evidence emphasizes the functional impacts of host microbiome on the etiopathogenesis of autoimmune diseases, including rheumatoid arthritis (RA). However, there are limited mechanistic insights into the contribution of microbial biomolecules especially microbial peptides toward modulating immune homeostasis. Here, by mining the metagenomics data of tonsillar microbiome, a deficiency of the encoding genes of lantibiotic peptides salivaricins in RA patients is identified, which shows strong correlation with circulating immune cells. Evidence is provided that the salivaricins exert immunomodulatory effects in inhibiting T follicular helper (Tfh) cell differentiation and interleukin‐21 (IL‐21) production. Mechanically, salivaricins directly bind to and induce conformational changes of IL‐6 and IL‐21 receptors, thereby inhibiting the bindings of IL‐6 and IL‐21 to their receptors and suppressing the downstream signaling pathway. Finally, salivaricin administration exerts both prophylactic and therapeutic effects against experimental arthritis in a murine model of RA. Together, these results provide a mechanism link of microbial peptides‐mediated immunomodulation.

## Graphical abstract

The present study uncovers that the microbiome‐derived lantibiotics salivaricins directly act on IL‐6 and IL‐21 receptors and suppress  the abnormal activation of Tfh cell and overproduction of IL‐21, thus maintaining host immune homeostasis. The deficiency of salivaricins in the tonsillar microbiome of autoimmune diseases like rheumatoid arthritis may reduce the capacity for proper modulation of immune responses, thereby underlying autoimmune disorders.

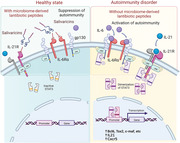

## 1 Introduction

The human microbiomes are well appreciated for their indispensable role in immune development. The immune system and the microbiota are two components that influence one another to orchestrate host homeostasis as well as to maintain a stable microbial community.^[^ ^1^ ^]^ Given the well‐recognized roles of microbiome in setting the systemic immune tone, perturbation of the healthy microbial community, termed dysbiosis, has been linked to the pathophysiology of numerous autoimmune diseases, including Type 1 diabetes, multiple sclerosis, and rheumatoid arthritis (RA).^[^ ^1^, ^2^ ^]^ Some autoimmune conditions like RA have required the presence of microbes for disease onset.^[^ ^3^ ^]^

RA is a systemic autoimmune disease characterized by the invasion of joints by proinflammatory immune cells and immune‐complexes formed from autoreactive antibodies.^[^ ^4^ ^]^ Multiple studies using inflammatory arthritis models have demonstrated that germ‐free or antibiotics‐treated mice could not develop arthritis unless particular bacteria are introduced (e.g., segmented filamentous bacteria, *Lactobacillus Bifidus*, or *Prevotella copri*),^[^ ^5^ ^]^ indicating an indispensable role for microorganisms in RA initiation. In support, clinical studies have shown that RA patients exhibit microbial dysbiosis in multiple body sites, including the gut, the oral cavity, and lungs.^[^ ^6^ ^]^ Despite these findings, the molecular links between the microbiome and RA pathogenesis remain to be addressed.

Microbial macro‐molecules and small‐molecule metabolites serve as an additional layer of communication between the host immunity and microbiome.^[^ ^7^ ^]^ The identification of specific microbiome‐derived biomolecules and their effect on the immune system have provided mechanistic insight into the immune cell‐microbiome co‐regulation. Microbial metabolites such as short‐chain fatty acids and bile acids, have been shown to modulate immune responses through direct regulation of regulatory T (Treg) and/or T helper 17 (Th17) cells.^[^ ^7^, ^8^ ^]^ However, there are relatively few mechanistic insights into the contribution of microbial macro‐molecules such as microbial peptides toward modulating immune homeostasis.

Previously, by profiling the metabolic potential of the tonsillar microbiome, we found that the biosynthesis and transport of lantibiotics were deficient in RA tonsils.^[^ ^9^ ^]^ Lantibiotics are polycyclic antimicrobial peptides containing lanthionine and/or *β*‐methyllanthionine residues produced by bacteria including species of *Streptococcus* and *Lactobacillus*.^[^ ^10^ ^]^ They are among the most promising candidates for future antimicrobials due to their capacity to inhibit the growth of clinically significant pathogens including multidrug‐resistant *Staphylococci*, *Streptococci*, *Enterococci*, and *Clostridia*.^[^ ^10^ ^]^ The diversity and potency of lantibiotics make them attractive candidates for translational application, and several are already in clinical trials.^[^ ^10^ ^]^ Besides the classical antibiotic use, they are now receiving increased attention as possible immune‐modulating agents.^[^ ^10^, ^11^ ^]^ The lantibiotic nisin Z is able to modulate host immune responses and mediate protective host immunity through similar mechanisms as natural host defense peptides, engaging multiple signal transduction pathways and growth factor receptors.^[^ ^12^ ^]^ Another lantibiotic peptide lancovutide (Moli1901) was shown to be a safe and effective therapy for the treatment of cystic fibrosis in a phase II clinical trial.^[^ ^13^ ^]^ These results suggest that the lantibiotic peptides may represent a new class of secreted bacterial molecules with immunomodulatory activities.

In the present study, we identified deficiency for lantibiotic peptides salivaricins in the tonsillar microbiome of RA patients. More importantly, we demonstrated the immunomodulatory effects of salivaricins, and determined the key receptors that mediated this process. Finally, animal experiments were performed to investigate the potential clinical applications of salivaricins in autoimmune diseases.

## 2 Results

### 2.1 Tonsillar microbiome‐Derived Lantibiotic Peptides Salivaricins are Correlated to Circulating Immune Cells

**Figure 1:**
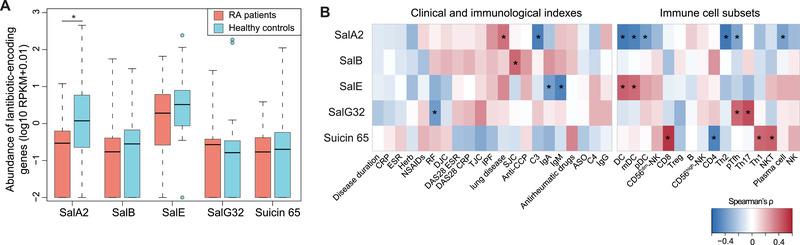
Tonsillar microbiome‐derived lantibiotic peptides salivaricins are deficient in RA patients and correlated to circulating immune cells. A) Boxplot showing the relative abundances of five lantibiotic genes (salivaricin A2, B, E, G32, and suicin 65) in the tonsillar microbiomes of rheumatoid arthritis (RA) patients and healthy controls. Boxes represented the interquartile range between the first and third quartiles and median (internal line). Whiskers denoted the lowest and highest values within 1.5 times the range of the first and third quartiles, respectively; and dots represented outlier samples beyond the whiskers. RPKM, reads per kilobase per million mapped reads. *n* = 32 for RA patients and *n* = 30 for healthy controls, Wilcoxon rank sum test, *P < 0.05. B) Heatmaps displaying the associations of lantibiotic‐encoding gene abundances from the tonsillar microbiome with RA‐related clinical and immunological indexes (left panel) and with circulating immune cell subsets (right panel). Spearman's rank correlation test, *P < 0.05.

Previously, by profiling the tonsillar microbiome of RA patients and healthy controls using a standard whole‐metagenome shotgun sequencing technology (Table S1, Supporting Information), we detected that the biosynthesis and transport of lantibiotics were deficient in RA tonsils.^[^ ^9^ ^]^ Here, we profiled these metagenomic sequencing data in‐depth to determine the genomic capacity of human tonsil‐associated microbes to produce lantibiotic peptides. Five lantibiotic‐encoding genes were identified from our metagenomic data by searching the antimicrobial peptide database (APD3: the antimicrobial peptide database as a tool for research and education),^[^ ^14^ ^]^ including salivaricin A2,^[^ ^15^ ^]^ B,^[^ ^15^, ^16^ ^]^ E,^[^ ^17^ ^]^ and G32,^[^ ^18^ ^]^ commonly synthesized by *S. salivarius*, and suicin 65,^[^ ^19^ ^]^ commonly synthesized by *S. suis* (Table S2, Supporting Information). Abundance of salivaricin A2 coding gene was significantly reduced in the tonsillar microbiomes of RA patients compared to that of healthy controls (Wilcoxon rank sum test, *P* = 0.03), while salivaricin B, E, G32, and suicin 65 were also less abundant in RA patients but not significant (**Figure** **1**A). Additionally, we analyzed the coding capacity of these lantibiotic peptides in the saliva, dental plaque, and gut microbiomes of RA patients.^[^ ^6c^ ^]^ Similarly, we found that salivaricin B from the saliva microbiome showed a downtrend (Wilcoxon rank sum test, *P* = 0.38) in naïve‐treated RA patients while became partial recovery to the healthy state in treated patients, whereas salivaricin A2, E2, and G32 were rarely detected in these niches (Figure S1, Supporting Information).

Subsequently, we explored correlations of lantibiotic peptides with RA clinical and immunological indicators. Despite that a limited number of associations were observed between lantibiotics and RA clinical indexes, we did detect strong associations of these lantibiotic‐encoding genes with host circulating immune cell subsets (Figure 1B). Specifically, the lack of salivaricin A2 was correlated with the increased proportions of precursor follicular helper T (pTfh), dendritic cells, and plasma cells (Figure 1B), all well‐known immune mediators leading to overproduction of pathogenic autoantibodies in RA.^[^ ^20^ ^]^ Collectively, these results support the notion that salivaricin peptides produced by tonsillar microbiome may function in immunomodulatory roles in RA.

### 2.2 Salivaricins Downregulate IL‐21 Production in Human PBMCs

**Figure 2:**
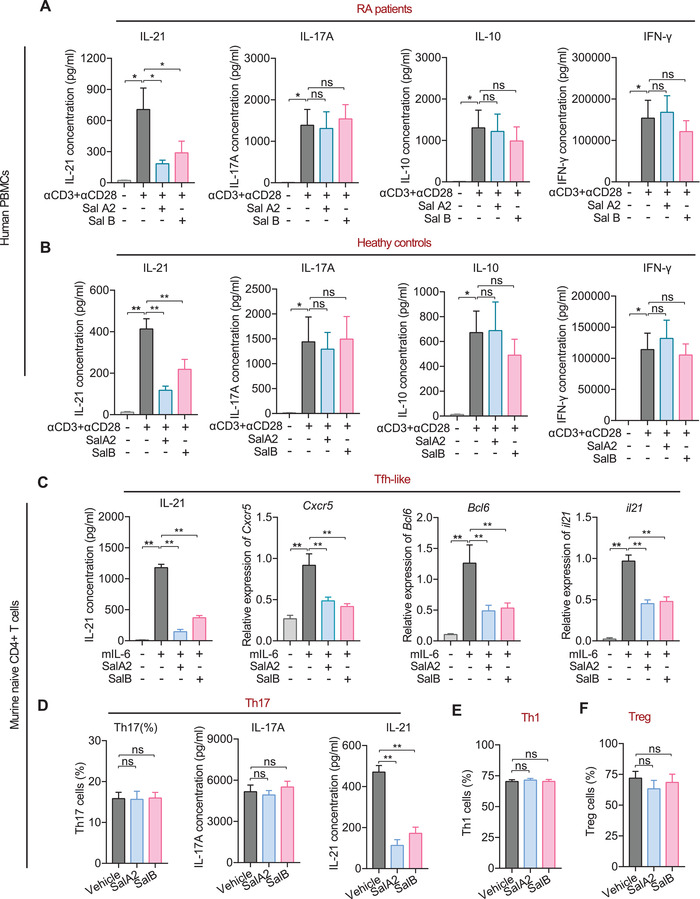
Salivaricins inhibit IL‐21 production and Tfh cell differentiation in vitro. A,B) PBMCs are isolated from RA patients (A, *n* = 9‐11) and healthy individuals (B, *n* = 7). Subsequently, these cells are cultured with activation by *α*CD3 and *α*CD28 antibodies and are exposed to salivaricin A2 or B for 3 days. The interleukin‐21 (IL‐21), IL‐17A, IL‐10, and IFN‐*γ* levels are measured by enzyme‐linked immunosorbent assay (ELISA). SalA2: salivaricin A2 (200 µg mL^−1^); SalB: salivaricin B (200 µg mL^−1^). C–F) Naïve CD4+ T cells from wildtype C57BL/6 mice are sorted by FACS and cultured in the presence of plate‐bound *α*CD3 and *α*CD28 antibodies, with or without salivaricin under Tfh‐like (C, *α*IFN‐*γ*+*α*IL‐4+*α*IL‐2+IL‐6), Th17 (D, *α*IFN‐*γ*+*α*IL‐4+IL‐6+TGF‐*β*+IL‐23), Th1 (E, *α*IL‐4+IL‐12), or iTreg (F, IL‐2+TGF‐*β*) cell differentiation conditions for 5 days. *Cxcr5*, *Bcl6*, and *il21* expression levels are measured by qPCR (c); the IL‐21 and IL‐17A levels are measured by ELISA kits (C,D); the proportions of Th17, Th1, and iTreg cells are assessed using flow cytometry (D–F). SalA2 = 50 µg mL^−1^; SalB = 50 µg mL^−1^. *n* = 12 for C; *n* = 10 for D; *n* = 11 for E; *n* = 8 for F. Data are expressed as the mean ± sem. Significance is assessed using one‐way ANOVA followed by Holm‐Sidak's multiple comparisons tests. *P < 0.05, **P < 0.01, ns, not‐significant.

To support investigations of the potential immunomodulatory effects of salivaricins, we chemically synthesized salivaricin A2 and B (Figure S2, Supporting Information) and performed in vitro experiments with peripheral blood mononuclear cells (PBMCs) isolated from both RA patients and healthy individuals. Treatment of cultured human PBMCs stimulated with *α*CD3 and *α*CD28 antibodies revealed that the salivaricin A2 or B peptides could significantly reduce the level of the immune regulatory cytokine interleukin‐21 (IL‐21), and the IL‐21 reducing effects of both peptides were dose‐dependent (Figure **2**A,B, and Figure S3, Supporting Information). No salivaricin‐related differences were observed in the levels of other cytokines including IL‐17A, IL‐10, IFN‐*γ*, TNF‐*α*, or IL‐6 (Figure S3, Supporting Information). IL‐21 is mainly produced by Tfh and Th17 cells, and this cytokine is known to regulate both germinal center (GC) B cell survival and plasma cell differentiation.^[^ ^21^ ^]^ Importantly, these in vitro results showing salivaricin A2‐ and B‐mediated reductions in the IL‐21 level of PBMCs are consistent with the negative correlations we detected in our RA patient clinical data between the capacity of the tonsillar microbiome to produce salivaricins and the proportions of circulating precursor Tfh and plasma cells.

### 2.3 Salivaricins Inhibit Tfh Cell Differentiation and Function

We next conducted in vitro differentiation experiments seeking to identify which cell subsets are directly targeted by salivaricin A2 and B. Naïve CD4^+^ T cells were isolated from wild‐type C57BL/6 mice and cultured with or without salivaricins under Tfh‐like (*α*IFN‐*γ*+*α*IL‐4+*α*IL‐2+IL‐6) or Th17 (*α*IFN‐*γ*+*α*IL‐4+IL‐6+TGF‐*β*+IL‐23) cell inducing conditions.^[^ ^22^ ^]^ First, we again found that salivaricin A2 and B both reduced the IL‐21 level in Tfh‐like cells in a dose‐dependent manner (Figure 2C and Figure S4A, Supporting Information). We also observed that salivaricin A2 and B led to dose‐dependent reductions in the expression levels of known Tfh‐polarized genes (e.g., *Cxcr5*, *Bcl6*, and *Il‐21*) compared to vehicle‐treated cells (Figure 2C and Figure S4B–F, Supporting Information). These results demonstrate that salivaricin A2 and B function as negative regulators of Tfh differentiation. Under Th17‐polarizing conditions, each of the tested salivaricins reduced the IL‐21 level, yet neither affected the IL‐17A level (Figure 2D and Figure S4G,H, Supporting Information). Thus, these salivaricin peptides can selectively suppress IL‐6 signaling, but do not obviously impact the TGF‐*β* and/or IL‐23 signaling pathways specific to Th17 conditions. Importantly, our control experiments showed that neither of the salivaricins affected the differentiation of Th1 (*α*IL‐4+IL‐12) or iTreg (IL‐2+TGF‐*β*) cells (Figure 2E,F and Figure S4I–L, Supporting Information).

### 2.4 Salivaricins Inhibit IL‐6R/IL‐21R‐STAT3 Signaling Pathway

**Figure 3:**
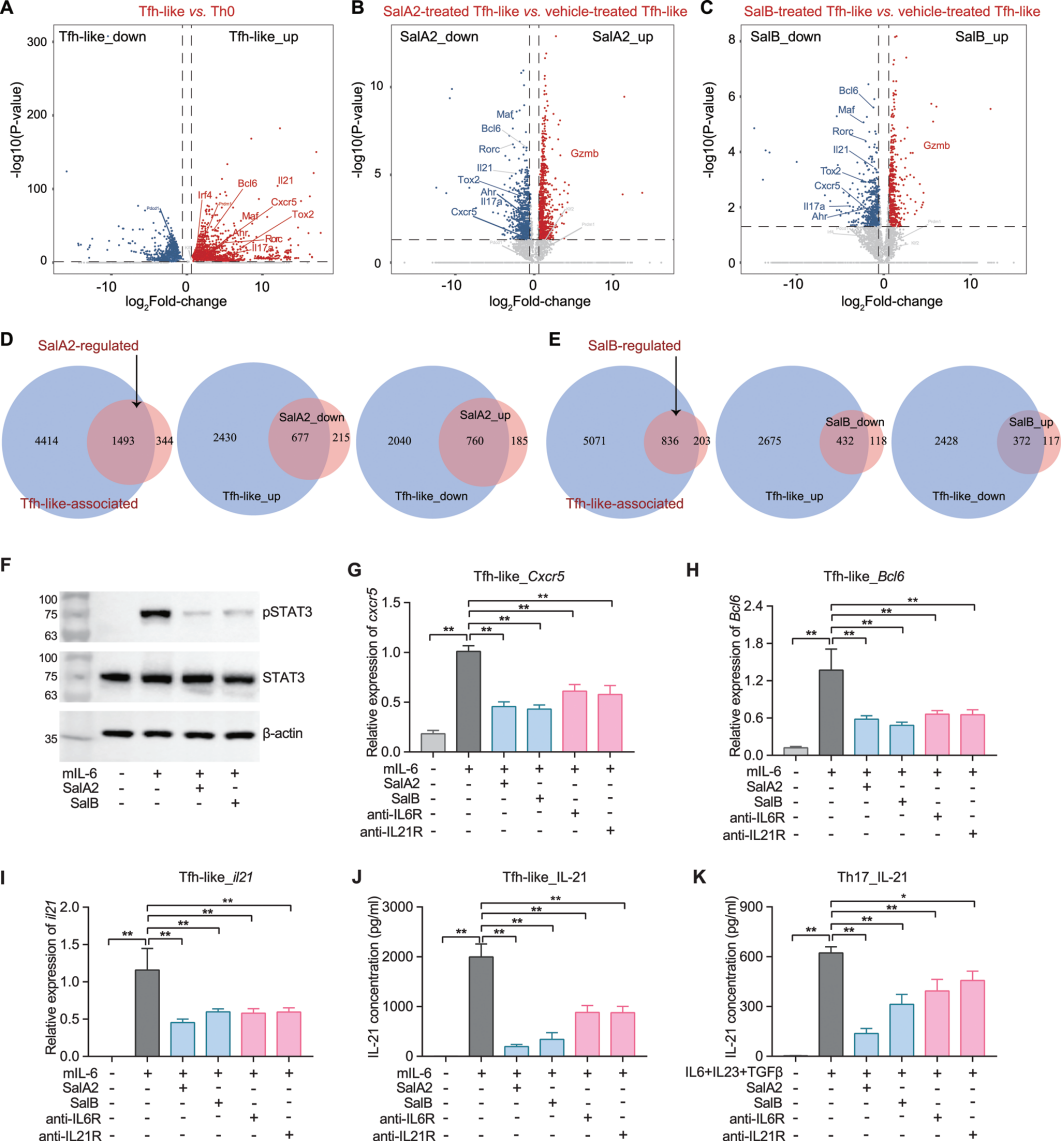
Salivaricins inhibit IL‐6R/IL‐21R‐STAT3 signaling pathway. A–C) Volcano plots of genes upregulated (red) or downregulated (blue) for 1.5‐fold or more in Tfh‐like relative to Th0 cells (A) or salivaricin‐treated relative to vehicle‐treated Tfh‐like cells (B,C) assessed by RNA sequencing (RNA‐seq) analyses. *n* = 5‐6; SalA2 = 50 µg mL^−1^; SalB = 50 µg mL^−1^. D,E) Venn diagrams showing the overlaps of gene profiles regulated between the salivaricin‐treated and vehicle‐treated Tfh‐like cells. sal‐regulated: differentially expressed genes between salivaricin‐treated and vehicle‐treated Tfh‐like cells; Tfh‐like‐associated: differentially expressed genes between Tfh‐like and Th0 cells. F) Assessment of phosphorylation level of signal transducer and activator of transcription 3 (STAT3) by western blot in Tfh‐like cells with or without salivaricin‐treatment. *n* = 3. G–J) Assessment of the expressions of *Cxcr5*, *Bcl6*, and *il21* (qPCR, (G–I)) and the IL‐21 level (ELISA, (J)) in Tfh‐like cells. SalA2 = 50 µg mL^−1^; SalB = 50 µg mL^−1^; anti‐IL6R = 50 µg mL^−1^; anti‐IL21R = 50 µg mL^−1^. *n* = 10. K) IL‐21 level (ELISA) in Th17 cells. *n* = 7–9. Data are expressed as the mean ± sem. Significance is assessed using one‐way ANOVA followed by Holm‐Sidak's multiple comparisons tests (B–F). *P < 0.05, **P < 0.01.

We further confirmed the negative regulation of salivaricin in driving Tfh‐associated transcriptional program by performing RNA sequencing (RNA‐seq) analyses (Figure **3**A–C). Overall, we observed that 81% of salivaricin‐regulated (differentially expressed genes between salivaricin‐treated and vehicle‐treated Tfh‐like cells) genes overlapped with Tfh‐like‐associated (differentiated expression genes between Tfh‐like and Th0 cells) genes, and 96% (677+760/1493 in salA2‐treated cells and 432+372/836 in salB‐treated cells) of these overlapped genes were negatively regulated by salivaricin treatment (Figure 3D,E). Given the dominant roles of IL‐6 and IL‐21 in shaping a Tfh‐like cell transcriptome,^[^ ^20^, ^23^ ^]^ we hypothesized that salivaricin A2 and B might somehow suppress IL‐6 and/or IL‐21 signaling. Supporting this idea, multiple Th17‐associated genes (e.g., *Il17a*, *Il17f*, *Ahr*, and *Rorc*) were also suppressed by salivaricins in Tfh‐like conditions (Figure 3A–C), further suggesting the inhibited role of salivaricins in IL‐6 and/or IL‐21 signaling.

Then we tested the activation of signal transducer and activator of transcription 3 (STAT3), a key molecule downstream of IL‐6 and/or IL‐21 signaling and also the most important signal transducer in promoting murine Tfh differentiation.^[^ ^20a^ ^]^ As expected, salivaricin A2 and B largely blocked IL‐6‐mediated STAT3 phosphorylation in Tfh‐like cells (Figure 3F and Figure S5A,B, Supporting Information). In the same line of evidence, both salivaricins can recapitulate the effects of anti‐IL‐6 receptor (IL‐6R) or anti‐ IL‐21 receptor (IL‐21R) neutralizing antibodies on Tfh‐like and Th17 cell differentiation and function (Figure 3G–K and Figure S5C,D, Supporting Information). Collectively, these results demonstrated that salivaricins could inhibit IL‐6R/IL‐21R‐STAT3 signaling pathway, thus rationalizing the observed effects of salivaricin on both Tfh cell differentiation and IL‐21 production.

### 2.5 Salivaricins Directly Bind to IL‐6 and IL‐21 Receptors

**Figure 4:**
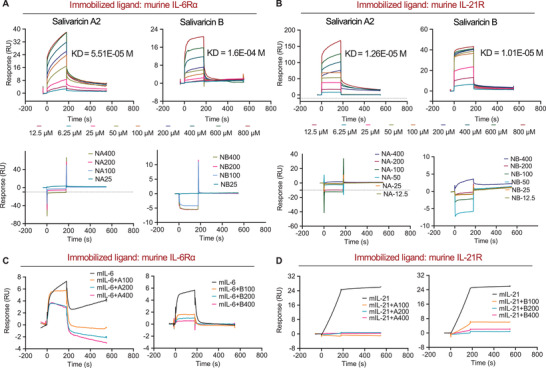
Salivaricins directly bind to IL‐6 and IL‐21 receptors. A) Surface plasmon resonance (SPR) sensorgram for the analyte salivaricin A2 or B binding to the immobilized murine IL‐6R, identifying dissociation constant (KD) values of 51.5 and 160 µM for salivaricin A2 and B with murine IL‐6R, respectively. IL‐6R*α*: IL‐6 receptor subunits alpha (20 nM); A: salivaricin A2 (6.25–800 µM); B: salivaricin B (6.25–800 µM); NA: negative control peptide for salivaricin A2 (25–400 µM); NB: negative control peptide for salivaricin B (25–400 µM). B) SPR sensorgram for the analyte salivaricin A2 or B binding to the immobilized murine IL‐21R, identifying KD values of 12.6 and 10.1 µM for salivaricin A2 and B with murine IL‐21R, respectively. IL‐21R: IL‐21 receptor (20 nM); A: salivaricin A2 (6.25–800 µM); B: salivaricin B (6.25–800 µM); NA: negative control peptide A (12.5–400 µM); NB: negative control peptide B (12.5–400 µM). C) Competitive inhibition assays of salivaricin A2 or B with murine IL‐6. IL‐6R*α*: 20 nM; mIL‐6: murine IL‐6 (50 nM); A: salivaricin A2 (100–400 µM); B: salivaricin B (100–400 µM). D) Competitive inhibition assays of salivaricin A2 or B with murine IL‐21. IL‐21R: 20 nM; mIL‐21: murine IL‐21 (1 nM). A: salivaricin A2 (100–400 µM); B: salivaricin B (100–400 µM).

Given the regulatory capacities of salivaricins in IL‐6R/IL‐21R signaling, we used cell‐free assays to test whether salivaricin A2 and B may interact with IL‐6R and/or IL‐21R. Pull‐down assays showed that salivaricin A2 and B, but not similarly sized negative control peptides, bound directly to murine IL‐6R subunit alpha (IL‐6R*α*); and only salivaricin B but not A2 bound directly to IL‐21R (Table S3, Supporting Information). Additional validation of direct bindings of salivaricins to murine IL‐6R*α* was performed using surface plasmon resonance (SPR), and the results demonstrated that salivaricins‐IL‐6R*α* interactions were concentration‐dependent (Figure **4**A). Plus, while pull‐down assay did not detect interaction between salivaricin A2 and IL‐21R, SPR experiments demonstrated that salivaricin A2 also interacts with murine IL‐21R (Figure 4B). It should be noted that, however, the detected binding affinities of the salivaricins to immobilized IL‐6R*α* and IL‐21R were 10^3^–10^5^ magnitude lower than the binding affinities of IL‐6 and IL‐21 to their receptors (Figure S6 and Table S4, Supporting Information). Despite having lower binding affinity to the respective receptors, the competitive inhibition experiments showed that the binding of IL‐6 to IL‐6R*α* or IL‐21 to IL‐21R could be partially or completely suppressed in the presence of salivaricin A2 or B, in a dose‐dependent manner (Figure 4C,D). These results indicated that salivaricins could inhibit the interactions between IL‐6 and IL‐21 with their receptors, therefore suppressing the IL‐6R/IL‐21R signaling.

**Figure 5:**
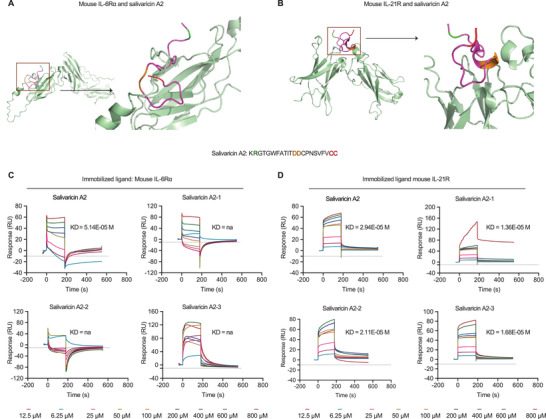
Identification of binding sites of salivaricin A2 to receptors. A,B) Computational approach is used to predict the potential amino acid sites of salivaricin A2 binding to mouse IL‐6R*α* and IL‐21R, identifying five residues according to the results generated by PISA (Proteins, Interfaces, Structures and Assemblies）. C) SPR sensorgram for the bindings of salivaricin A2 mutants (12.5–800 µM) to the immobilized murine IL‐6R (20 nM). The KD values of salivaricin A2 is 55.1 µM, while salivaricin A2‐1 (mutation at the 2nd arginine residue), salivaricin A2‐2 (mutations at the 12th and 13th asparagine residues), and A2‐3 (mutations at 21st and 22nd cysteine residues) showed not‐available KD values to murine IL‐6R*α*. D) SPR sensorgram for the bindings of salivaricin A2 mutants (12.5–800 µM) to the immobilized murine IL‐21R (20 nM), identifying KD values of 29.4 µM for salivaricin A2, 13.4 µM for salivaricin A2‐1, 21.1 µM for salivaricin A2‐2 and 16.8 µM for salivaricin A2‐3 to murine IL‐21R.

Next, we set to identify the key amino acid sites of salivaricins that mediate the bindings to IL‐6R and/or IL‐21R. First, computational approach was used to predict the potential amino acid sites on the peptides (Figure**5**A,B). We then constructed five mutants according to the results generated by PISA (Proteins, Interfaces, Structures, and Assemblies) and validated their binding affinities by SPR. Results showed that salivaricin A2 peptides with mutations at the 2nd arginine residue (salivaricin A2‐1), at the 12nd and 13th asparagine residues (salivaricin A2‐2), and at the 21st and 22nd cysteine residues (salivaricin A2‐3) all had impaired binding affinities to IL‐6R (Figure 5C), yet remained similar binding affinities to IL‐21R (Figure 5D). In addition, these mutants had similar secondary structures with salivaricins A2 (Figure S7, Supporting Information), indicating that these sites were not critical for the conformation of salivaricin A2, but responsible for its associations with IL‐6R. However, the predicted sites of salivaricin B showed little effect in the binding affinities to IL‐6R or IL‐21R (Figure S8, Supporting Information), indicating a gap in predicting and understanding of functionality of salivaricin B, which would only be addressed in future studies.

### 2.6 Salivaricins Induce Conformational Changes of IL‐6 and IL‐21 Receptors

**Figure 6:**
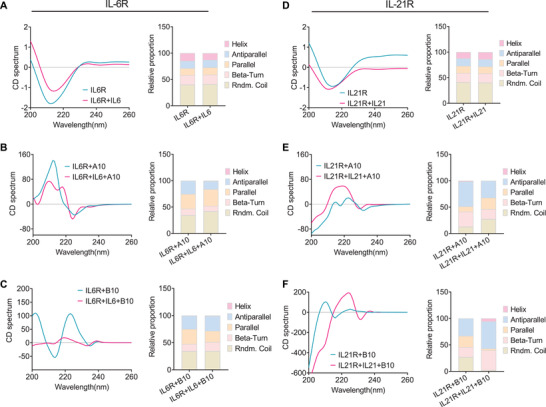
Salivaricins induce conformational changes in IL‐6 and IL‐21 receptors. A–C) The circular dichrois (CD)） spectrum and the statistical proportions of different secondary structures (e.g., alpha‐helix, beta‐barrels, and random coils) of IL‐6R*α* in the presence or absence of IL‐6 (A) and salivaricins with or without IL‐6 (B,C). D–F) The CD spectrum and the statistical proportions of different secondary structures of IL‐21R in the presence or absence of IL‐21 (D) and salivaricins with or without IL‐21 (E,F). mIL‐6R: 0.1 mg mL^−1^; SalA2: 10 mg mL^−1^; SalB: 10 mg mL^−1^ mIL‐6R: 0.1 mg mL^−1^. mIL‐21R: 0.1 mg mL^−1^; SalA2: 10 mg mL^−1^; SalB: 10 mg mL^−1^; mIL‐21: 0.1 mg mL^−1^.

As the salivaricins had much lower binding affinities to the respective receptors, we then hypothesized that the peptides did not directly compete for binding with IL‐6 or IL‐21 but rather induce changes in the functions of receptors. Given that the receptor structure is critical to the ligand‐receptor engagement, we detected the secondary structures of IL‐6R*α* and IL‐21R in the presence of salivaricins by using circular dichroism (CD) spectroscopy. As shown in Figure **6**, the CD spectrum and the statistical proportions of different secondary structures (e.g., alpha‐helix, beta‐barrels, and random coils) of IL‐6R*α* or IL‐21R remained unchanged following IL‐6 or IL‐21 binding (Figure 6A,D), and in contrast, salivaricins dramatically altered IL‐6R*α* and IL‐21R structures in a dose‐dependent manner (Figure 6B,C,E,F, and Figure S9, Supporting Information). Additionally, the structures of the two receptors were also changed in response to salivaricins with the presence of IL‐6 or IL‐21 (Figure 6B,C,E,F), providing evidence that the structural changes of IL‐6 and IL‐21 receptors are involved in salivaricins’ competition with interleukins.

### 2.7 Salivaricins Exert Anti‐Arthritic Effects in Experimental Arthritis

**Figure 7:**
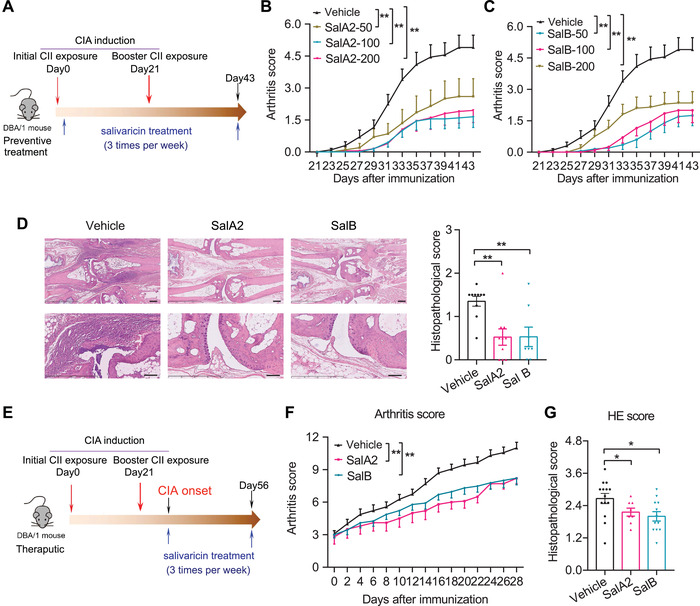
Salivaricins confer protection against experimental arthritis in mice. A) Experimental design schematic for testing salivaricins as a prophylactic regimen with the collagen‐induced arthritis (CIA) mouse model. B,C) Clinical arthritis scores in CIA mice with or without intral‐orally administration of salivaricin A2 (B) or B (C). SalA2: salivaricin A2 (50, 100, or 200 µg per mice); SalB: salivaricin B (50, 100, or 200 µg per mice); *n* = 20 for each group. D) Histopathological scoring of the paws showing significantly decreased inflammation in salivaricins‐treated mice. Vehicle, *n* = 10; SalA2 (100 µg per mice), *n* = 10; SalB (100 µg per mice), *n* = 8. Bar = 250 mm and 100 um for the upper and lower panel, respectively. E) Experimental design schematic for testing salivaricins as a therapeutic regimen with the CIA mouse model. F) Clinical scores of arthritis in the indicated groups. Vehicle, *n* = 23; SalA2 (100 µg per mice), *n* = 10; SalB (100 µg per mice), *n* = 23. G) Histopathological scoring of the paws. Vehicle = 14, SalA2 = 8, SalB = 12. Data are pooled from two independent experiments and expressed as mean ± sem. Significance determined using two‐way ANOVA followed by Tukey's multiple comparisons test (B,C,F) or Mann–Whitney U test (D,G), *P < 0.05, **P < 0.01.

Interventions with agents to modulate IL‐6/IL‐6R or IL‐21/IL‐21R have been shown to confer clinical benefit in patients with autoimmune diseases including RA.^[^ ^21^, ^24^ ^]^ To determine if salivaricins can suppress immune‐mediated pathological processes in vivo, we explored both prophylactic and therapeutic applications of salivaricins in collagen‐induced arthritis (CIA) model mice, a well‐characterized murine arthritis model. Prophylactic administration with salivaricin A2 or B before arthritis onset (see **Figure** **7**A for the experimental design) significantly decreased the incidence and severity of arthritis in the CIA mice, without affecting body weight (Figure 7B–D and Figure S10A–G, Supporting Information). Note that similar protective effects were observed for all three of the salivaricin administration routes (intra‐orally, intra‐peritonelly, or intra‐gastrically). We also administered salivaricins as a potential treatment after CIA model induction, and found that both salivaricin A2 and B significantly relieved disease severity and reduced inflammatory cell infiltration in the joints compared to the vehicle controls (Figure 7E–G). Thus, salivaricins have potential clinical applicability, both for protecting against RA development and for treating RA.

**Figure 8:**
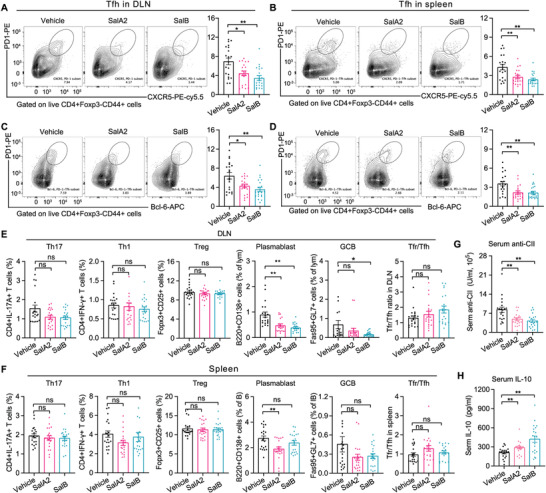
Salivaricins inhibit immune responses in CIA mice. A–D) Representative flow cytometry plots with graphs showing frequencies of Tfh cells (CD4^+^CD44^+^CXCR5^+^PD1^hi^Bcl6^+^) in the draining lymph nodes (DLNs) and spleens of the indicated groups. *n* = 20 per group. E,F) Graphs showing frequencies of Th17 cells (CD4^+^IL‐17A^+^IFN‐*γ* ^−^), Th1 cells (CD4^+^IFN‐*γ* ^+^IL‐17A^−^), Treg cells (CD4^+^CD25^+^Foxp3^+^), plasmablast (B220+CD4^−^CD138^+^), and GC B cell (B220+CD4^−^Fas95^+^GL‐7^+^) and ratio of Tfr to Tfh cells in the DLNs and spleens of the indicated groups. *n* = 20 per group. G,H) Serum concentrations of anti‐IgG collagen type II (CII) antibody and IL‐10. *n* = 20 per group, SalA2 = 100 µg per mice and SalB = 100 µg per mice treated by intraorally. Data is pooled from two independent experiments and expressed as mean ± sem. Significance determined using Mann–Whitney test, *P < 0.05, **P < 0.01. ns: not‐significant.

We also explored which immune effector molecules may contribute to the observed anti‐arthritic efficacy of salivaricins by profiling serum cytokine levels and lymphocyte subsets in draining lymph nodes (DLN) and spleens of the animals at the end of the prophylactic regime experiment. Salivaricins markedly decreased the proportions of Tfh cells in both DLNs and spleens (**Figure** **8**A,B). We also detected a significant reduction of Tfh cells expressing the aforementioned Tfh polarizing transcription factor Bcl6 (Figure 8C,D). In addition, the frequencies of Th17, plasmablast, and GCB cells also showed trends of decrease in the salivaricins‐treated mice, while the proportions of Th1 and Treg cells remained unchanged (Figure 8E,F). Similar to Tfh cells, Tfr cells were also reduced in the salivaricin‐treated mice (Figure S10H,I, Supporting Information), while the ratio of Tfr to Tfh cells showed increased tendency (Figure 8E,F). Consistently, salivaricins‐treated mice showed significantly reduced titers of serum autoantibody anti‐collagen type II (CII) and significantly increased serum IL‐10 levels (Figure 8G,H). There was no change in the IL‐6 level (Figure S10J, Supporting Information). However, serum IL‐21 in CIA mice was overall very low, which may underlie the fact that we found trend of decrease but no statistical significance in IL‐21 level in salivaricin‐treated mice (data not shown). Together, these data suggest that salivaricin may act as a promising target in the management of overproduction of pathogenic autoantibodies in autoimmune diseases including RA.

## 3 Discussion

In this study, we revealed an immunomodulatory role of lantibiotic peptides salivaricins in autoimmune disease, demonstrating that salivaricins beneficially modulate host immunity by directly binding to and inhibiting IL‐6 and IL‐21 receptors.

Lantibiotic peptides are the most extensively studied antimicrobial peptides that are derived from bacteria. They are now receiving increased attention as potential clinical antimicrobials and as possible immune‐modulating agents. The identification of lantibiotics with immunomodulatory activity other than antimicrobial effects will enlarge their possibilities for applications. Salivaricins are produced by certain strains of *Streptococcus salivarius*, which almost exclusively reside in the human oral cavity. Salivaricins have been useful in the development of novel antibacterial therapies as an alternative to conventional antibiotics.^[^ ^10^ ^]^ Here, we investigated in‐depth the immunomodulatory effect of salivaricins, and demonstrated that salivaricin A2 and B beneficially modulate host immunity by inhibiting Tfh cell differentiation and IL‐21 production. Consistent with the dominant roles of Tfh cells and IL‐21 in promoting GCB cell survival, plasma cell differentiation, and antibody production,^[^ ^20^, ^21^ ^]^ we detected reduction of plasmablast, GCB cells, and autoantibody levels along with the downregulated Tfh cells in salivaricin‐treated CIA mice. Therefore, we speculate that the deficiencies of salivaricins in RA tonsils reduce the capacity for proper modulation of local immune responses and autoantibody production, by which they are able to regulate autoimmune responses at distal sites involving the synovial joints. The present study, together with previous reports showing that gut microbiota can remotely regulate experimental arthritis by driving the induction and egress of gut Tfh cells,^[^ ^5^ ^]^ suggests that the disease‐relevant T cell subsets or autoantibodies in RA may be initially generated (and proliferated) outside of the joints. Nevertheless, we do not have direct evidence to support the notion that pathological T cells primed at the oral cavity migrate into the joints.

The cytokine milieu is critical for Tfh cell differentiation. IL‐6/IL‐6R and/or IL‐21/IL‐21R are indispensable regulators that affect the differentiation and function of Tfh cells.^[^ ^25^ ^]^ Previous studies have reported that Tfh cell induction was impaired in IL‐6^−/−^ or IL‐6R^−/−^ mice.^[^ ^26^ ^]^ Similarly, there was a defect in the generation of Tfh cells in IL‐21^−/−^ or IL‐21R^−/−^ mice.^[^ ^27^ ^]^ In addition, IL‐21 or IL‐21R deficient naïve CD4^+^ T cells showed significantly reduced Bcl6 expression in the presence of IL‐6.^[^ ^23a^ ^]^ IL‐21 is induced by IL‐6 in activated T cells, and can in turn signal to reinforce its own production.^[^ ^28^ ^]^ In the present study, we demonstrate that salivaricin A2 and B can directly bind to IL‐6R and IL‐21R. Although salivaricin A2 or B bind to IL‐6R*α* or IL‐21R with much lower affinities than that of IL‐6 or IL‐21, they are able to change the structures of IL‐6R*α* and IL‐21R. It is likely that the altered conformations of IL‐6R*α* and IL‐21R in response to salivaricin‐binding may be responsible for the salivaricins’ competition with interleukins and the reduced interactions of IL‐6/IL‐6R and IL‐21/IL‐21R. Consequently, salivaricins can inhibit the IL‐6R/IL‐21R‐STAT3 signaling, thereby suppressing Tfh cell differentiation and IL‐21 production. Although both salivaricin A2 and B can bind to and inhibit IL‐6R, it is noted that salivaricin A2 had slightly higher affinity to IL‐6R than salivacicin B, which may be responsible for the better inhibition in IL‐21 production. Moreover, the abundance of salivaricin A2 coding gene was more abundant and frequent than salivaricin B in healthy individuals, making it more suitable for health concerns in the future.

Furthermore, IL‐6/IL‐6R and IL‐21/IL‐21R are important contributors to the development of multiple autoimmune disorders characterized by overproduction of pathogenic autoantibodies.^[^ ^21^, ^24^ ^]^ Studies have reported that knockout of IL‐6 or IL‐21R protected mice from experimental arthritis in various models.^[^ ^24^, ^29^ ^]^ In light of these discoveries, numerous agents which target components of these two signaling pathways have drawn much biomedical attention.^[^ ^21^, ^24^ ^]^ For instance, monoclonal antibodies against IL‑6R (e.g., Tocilizumab) have been developed for the treatment of RA.^[^ ^24^ ^]^ Here, we confirmed the ability of salivaricin A2 and B to modulate host immunity in vivo, as evidenced by the reduced autoantibody production and Tfh cells of murine RA model, and detected both prophylactic and therapeutic efficacy against experimental arthritis. Therefore, establishing strategies for manipulating oral microbiome with salivaricins or related probiotics (e.g, *S. salivarius*) may represent a useful strategy for developing therapeutics against overproduction of pathogenic autoantibodies in autoimmune diseases including RA. Our study paves the way for novel regime of treatment for RA and perhaps other autoimmune disorders.

Mucosal niches including the gut and the oral are viewed as likely extra‐articular origins for RA development.^[^ ^3^, ^30^ ^]^ Multiple studies have proposed that interactions between the gut and oral mucosal immune system and an aberrant local microbiome might have a causal role in the development of RA.^[^ ^5^ ^]^ The tonsillar microbiome is an example of a lymphoid organ‐attached community.^[^ ^31^ ^]^ The close contacts of such communities with mucosa enable their direct interaction with the immune system.^[^ ^31^, ^32^ ^]^ Focal infections in the tonsils have long been implicated in RA pathogenesis, albeit without well‐defined mechanisms.^[^ ^30^, ^33^ ^]^ Supporting this idea, our data identified that lantibiotic peptides salivaricins were deficient in the tonsillar microbiome of RA patients. However, the expression of salivaricin in healthy subjects was too low for liquid chromatography‐mass spectrometry detection. Previous study has detected the presence of salivaricin peptides in human saliva using the induction assay,^[^ ^34^ ^]^ but only in subjects in which significant levels of salivaricin‐producing strain (eg., *S. salivarius* K12) colonization were achieved. In their study, the lowest concentration of *S*. *salivarius* to detect salivaricin appeared to be 8*10^5^ CFU per mL.^[^ ^34^ ^]^ By contrast, the *S. salivarius* levels were only 2.6*10^5^ CFU per mL in healthy subjects,^[^ ^34^ ^]^ thus making it hard to detect the presence of salivaricin without supplementation of salivaricin‐producing strain. In addition, currently there are no ELISA‐kits or antibodies available, we, therefore, have to rely on metagenomic data for inferring abundances of bacterial peptides, a common practice in microbiome research.

More importantly, we observed that the lack of salivaricin A2 at tonsils was correlated with the increased proportions of pTfh, dendritic cells, and plasma cells in the circulating, which is consistent with the immunomodulatory effects of salivaricin A2 in inhibiting Tfh cell differentiation and function. In addition, salivaricin B also showed negative correlations with pTfh cells but did not reach significance, the trend is consistent with the negative regulation of salivaricin B in Tfh cells. Presumably, the relatively small sample size and/or low expression of salivaricin B in human might be a contributing factor. Moreover, salivaricins are known to exert antimicrobial activities selectively against oral and upper‐respiratory‐tract pathogenic bacteria including *S. pyogenes* and *S. dysgalactiae*,^[^ ^10^, ^15^, ^35^ ^]^ which are expectedly enriched in RA tonsillar microbiome^[^ ^9^ ^]^ and known to trigger autoimmune responses in the joints and the heart.^[^ ^36^ ^]^ Therefore, we speculate that the potential therapeutic advantage of salivaricins may stem from its dual action involving an immunoregulatory mechanism and an antimicrobial activity. In addition, although salivaricin A2 was positively correlated with lung disease, whether salivaricin A2 is a risky factor to lung health or causally implicated in lung diseases needs to be studied further.

In summary, the major findings of the present study are the identification of immunomodulatory roles of lantibiotics salivaricins in modulating host autoimmunity and the recovery of key receptors that mediate this process.

## 4 Experimental Section

### Participant Enrollment

Adult patients (n = 32) diagnosed with RA according to the American College of Rheumatology/European League Against Rheumatism (ACR/EULAR) 2010 classification criteria^[^ ^37^ ^]^ were recruited from Peking University People's Hospital, Beijing, China. In the RA group, 37.5% (12/32) patients were treatment‐naïve. Healthy volunteers (n = 30) with no history of inflammatory arthritis and rheumatic diseases were enrolled. All the participants had not taken antibiotic treatment or probiotic supplements in the three months prior to sample collection. Informed consent was obtained from all subjects. This study was approved by Peking University People's Hospital Ethics Committee (2101000499). Detailed information of the cohort was given in Tables S1.

### Whole‐Metagenome Shotgun Sequencing and Bioinformatic Analyses

The tonsillar microbiota samples were collected by following the procedure of Human Microbiome Project (https://hmpdacc.org/doc/HMP_MOP_Version12_0_072910). Genomic bacterial DNA was extracted using the MoBio PowerSoil DNA Isolation Kit 12888‐100 protocol (MoBio Laboratories). The samples were placed at −80 °C less than two months before DNA extraction. The fresh genomics DNA samples (RA = 32 and HC = 30) were mechanically fragmented to ≈400 bp with Bioruptor Pico (Diagenode, Belgium). A magnetic beads‐based method was used for DNA fragments selection following a standard protocol (Agencourt AMPure XP). Libraries were prepared by using the NEBnext® Ultra II DNA Library Prep Kit for Illumina® (New England BioLabs). The Illumina HiSeq X platform was then used for 2 × 150 bp paired‐end whole‐metagenome sequencing. A *de novo* gene catalogue was constructed based on the metagenomic data from the tonsillar samples of all individuals. A non‐redundant gene catalogue was generated after removing the redundancy genes.

The lantibiotics and their encoding genes from the metagenomic data are identified based on integrating the antimicrobial peptide database (APD3)^[^ ^14^ ^]^ and literature search with a threshold of >95% similarity. For the APD3 database, only lantibiotics that sourced from the organisms of *Streptococcus*, *Lactococcus*, and *Lactobacillus* were selected for further analysis. And a literature search was performed by searching the PubMed database using keywords “lantibiotics”, or “salivaricin”. This procedure identified 11 additional salivaricins from several strains of *Streptococcus* spp. and *Lactobacillus salivarius*. The detailed information on lantibiotics inferred in this study was shown in Table S2, Supporting Information.

### The Chemosynthesis of Peptides

Salivaricin A2 (KRGTGWFATITDDCPNSVFVCC),^[^ ^15b^ ^]^ salivaricin B (GGGVIQTISHECRMNSWQFLFTCCS),^[^ ^16^ ^]^ negative control peptide A (CCVFVSNPCDDTITAFWGTGRK), and negative control peptide B (SCCTFLFQWSNMRCEHSITQIVGGG) were synthesized by solid‐phase techniques on a CS336X Peptide Synthesizer (Cs Bio, USA) in RoYo Biotech Co., Ltd (Shanghai, China). The reverse peptides of salivaricin A2 and B were chosen as their negative control peptides according to previous study.^[^ ^38^ ^]^ The peptides were purified by high‐performance liquid chromatography (Shimadzu Corp., Japan) with a purity of more than 96% and identified by Liquid chromatography‐mass spectrometer (Shimadzu Corp., Japan). The endotoxin unit was tested. Detailed information on the two salivaricins was given in Figure S2, Supporting Information.

### PBMCs Preparation and Culture

PBMCs were isolated from the blood of RA patients and healthy donors as previously described.^[^ ^20^ ^]^ Briefly, after gradient centrifugation by using lymphocyte separation medium (TBD science), mononuclear cells were collected, washed in RPMI 1640 medium (Gibco, ThermoFisher Scientific), and adjusted to 10^6^ cells mL^−1^ in 1640 supplemented with 50 IU mL^−1^ penicillin (Gibco, ThermoFisher Scientific), 50 µg mL^−1^ streptomycin (Gibco, ThermoFisher Scientific), and 10% fetal bovine serum (FBS, Gibco, ThermoFisher Scientific). PBMCs (10^6^ cells mL^−1^) were seeded in a 48‐well tissue culture plates (Corning, New York, USA), and then co‐incubated with anti‐CD3 (2 µg mL^−1^) plus anti‐CD28 (2 µg mL^−1^) antibody (biogems) with or without salivaricin A2 (12.5, 50, or 200 µg mL^−1^) or salivaricin *B* (12.5, 50, or 200 µg mL^−1^)^[^ ^12^ ^]^ at 37 °C in air with 5% CO_2_. After 66 h culturing, the supernatants were collected, clarified by centrifugation (3000 rpm, 10 min, room temperature). Cytokines were measured by enzyme‐linked immunosorbent assay (ELISA) kits for IL‐10, IL‐17A, IL‐21, IFN‐*γ*, TNF‐*α*, and IL‐6 according to the manufacturer's instructions (Multisciences, Hangzhou, China). Informed consent was obtained from all subjects. This study was approved by Peking University People's Hospital Ethics Committee.

### In Vitro T Cell Differentiation

Naive CD4^+^CD25^−^CD44^low^CD62L^hi^ T cells form peripheral (pooled inguinal, popliteal, axillary, and submandibular) lymph nodes of wild‐type C57BL/6 mice (6–8 weeks old) were FACS‐sorted and stimulated with the plate‐bound anti‐CD3 (2 µg mL^−1^) and anti‐CD28 (2 µg mL^−1^). The activated CD4^+^ T cells were cultured with anti‐IFN*γ* (XMG1.2, 10 µg m^−1^) and anti‐IL‐4 (11B11, 10 µg mL^−1^) for Th0 cells; with mIL‐6 (40 ng mL^−1^) plus anti‐IFN*γ* (10 µg mL^−1^), anti‐IL‐4 (10 µg mL^−1^) and anti‐IL‐2 (15 µg mL^−1^) for Tfh‐like cell differentiation; with hTGF*β* (0.5 ng mL^−1^), mIL‐6 (40 ng mL^−1^), mIL‐23 (50 ng mL^−1^) plus anti‐IFN*γ* (10 µg mL^−1^) and anti‐IL‐4 (10 µg mL^−1^) for Th17 cell differentiation; with mIL‐12 (5 ng mL^−1^) and anti‐IL4 (10 µg mL^−1^) for Th1 cell differentiation; with hTGF*β* (2 ng mL^−1^) with IL‐2 (50U mL^−1^) for iTreg cell differentiation as previously described.^[^ ^22^ ^]^ Then, these cells were treated with or without salivaricin A2 (12.5, 50, or 200 µg mL^−1^), B (12.5, 50, or 200 µg mL^−1^), anti‐IL‐6R neutralizing antibody (15A7; BioXcell, 50 µg mL^−1^), or anti‐IL‐21R neutralizing antibody (4A9; BioXcell, 50 µg m^−1^). All in vitro polarization assays were carried in complete RPMI 1640 with 10% FBS (final volume of 200 µL) in flat‐bottom 96‐well plates (Corning), and the cytokines and antibodies mentioned above were purchased from PeproTech, biogems, or BioXcell. On day 3, cells were expanded with additional medium and half‐concentration of cytokines. On day 5, cells were harvested and assayed for T cell subsets or reserved under −80 °C until DNA extraction and further assay. Cytokines (IL‐21, IL‐17A, IL‐10, and INF‐*γ*) in the supernatant were measured by ELISA kits.

### Real‐Time Quantitative PCR (qPCR)

For comparison of gene expression, total RNA from in vitro polarized Th0 or Tfh‐like cells was extracted with RNAprep Pure Micro Kit (TIANGEN Biotech Co., Ltd) according to the manufacturer's instructions. Oligonucleotide, Revertaid reverse transcriptase, and RiboLock RNase Inhibitor (Invitrogen) were used to generate cDNA. Gene expressions were examined using the AceQ qPCR SYBR Green Master Mix (Vazyme Biotech). For qPCR analysis, the data shown were normalized to the expression of reference gene Actb. The primers were listed in Table S5, Supporting Information.

### RNA Sequencing (RNA‐seq) Analyses

Total cellular RNA was extracted from the Th0, vehicle‐treated or salivaricin‐treated Tfh‐like cells, then processed for library preparation and sent for 2 × 150 bp paired‐end sequencing on an Illumina Novaseq 6000 (LC‐Bio Technology CO., Ltd) following the vendor's recommended protocol. RNA‐sequencing reads were aligned to the murine reference genome (https://ftp.ensembl.org/pub/release‐101/fasta/‐mus_musculus/dna/) using HISAT2 software (https://daehwankimlab.github.io/hisat2/,version:hisat2‐2.0.4). The mapped reads were assembled using StringTie (https://ccb.jhu.edu/software/stringtie/,version:stringtie‐1.3.4), and then all transcriptomes were merged to reconstruct a comprehensive transcriptome using gffcompare software (https://ccb.jhu.edu/software/stringtie/gffcompare.shtml,version:gffcompare‐0.9.8.). After the final transcriptome was generated, StringTie and ballgown (https://www.bioconductor.org/packages/release/bioc/html/ballgown.html) were used to estimate the expression levels of all transcripts calculated as FPKM (FPKM = (total_exon_fragments/mapped_reads(millions) × exon_length(kB))). Differentially expressed genes (DEGs) were identified by at least 1.5 fold change and *P* value < 0.05 (DESeq2 R package, https://www.bioconductor.org/packages/release/bioc/html/‐DESeq2.html).

### Western Blot

Cells from in vitro polarized Th0 or Tfh‐like cells were lysed using RIPA lysis buffer (Sigma‐Aldrich, St Louis, MO, USA) supplemented with 1% protease inhibitor cocktail and 5% phosphatase inhibitor (Roche, Basel, Switzerland. Protein concentrations were determined using BCA protein assays (Pierce, Rockford, IL, USA). Cell lysates were separated by SDS‐PAGE gels and electrotransferred onto polyvinylidene difluoride (PVDF) membranes (GE Healthcare, Buckinghamshire, UK), then probed with primary antibodies and subsequently HRP‐labeled secondary antibodies. Signals were detected by ChemiDoc MP (BIO RAD). Immunoblotting was performed using standard protocols. *β*Actin was used as an internal control throughout. The antibodies against pSTAT3 (Tyr705) and STAT3 (79D7) were obtained from Cell Signaling Technology.

### Protein Pull‐Down Assay

The recombinant mouse IL‐6 receptor subunit alpha protein with the Fc region of mouse IgG2a (Fc‐IL‐6R*α*, abcam) and mouse IL‐21 receptor protein with the Fc region of human IgG1 (IL‐21R‐Fc, Sino Biological) were commercially purchased. Fc‐IL‐6R*α* (0.5 µg) or IL‐21R‐Fc (0.5 µg) plus salivaricin A2 (5 µg), salivaricin B (5 µg), negative control peptides A2 (5 µg) or negative control peptides B (5 µg) were incubated in 1 mL of binding buffer (20 mM Tris‐HCl (pH 7.6), 200 mM NaCl, 1 mM EDTA, 2 × protease inhibitor (Thermo Scientific)) at 4 °C for 2 h on a rotation mixer. Subsequently, 10 µL of Protein A Agarose (Thermo Scientific) was added to the reaction system and incubated for an additional 2 h. After the incubation, a buffer containing 20 mM Tris‐HCl (pH 7.6), 200 mM NaCl, 1 mM EDTA, 0.6% Nonidet P‐40 (v/v), and 2 × protease inhibitor (Thermo Scientific) was used to wash the agarose 4 times, and a buffer containing 2% formic acid plus 50% acetonitrile was used to elute the agarose. Then, the samples were re‐suspended with 50 µL PBS, and detected the targeted proteins or peptides by LTQ‐Orbitrap Velos Mass Spectrometer, Q‐Exactive.

### SPR Experiments

The binding affinities of salivaricins to IL‐6R*α* or IL‐21R were assayed using the Biacore 8K instrument (GE Healthcare). Murine IL‐6R*α* (20 nM, abcam) or IL‐21R (20 mM, Sino Biological) were immobilized on a CM5 sensor chip using standard amine‐coupling at 25 °C with PBS‐P running buffer (GE Healthcare), as described previously.^[^ ^39^ ^]^ In the binding assays, a range concentrations of salivaricin A2 (6.25, 12.5, 25, 50, 100, 200, 400, 600, and 800 µM), salivaricin B (6.25, 12.5, 25, 50, 100, 200, 400, 600, and 800 µM), negative control peptides A2 (12.5, 25, 50, 100, 200, and 400 µM), negative control peptides B (12.5, 25, 50, 100, 200, and 400 µM), murine IL‐6 (0.003125–0.1 µM), or murine IL‐21(0.625–5 nM) containing 5% DMSO were serially injected into the channel to evaluate binding affinity. Regeneration was achieved by extended washing with NaOH (5 mm) after each sample injection. The dissociation constants (KD) of salivaricins were obtained by fitting the data sets to steady‐state affinity model using Biacore 8K Evaluation Software. For competitive inhibition experiments, murine IL‐6 or IL‐21 was injected into the channel in the presence or absence of salivaricin A2 or B.

### Computational Analysis of Binding Sites of Salivaricins with Receptors

Four complexes (salivaricin A2‐mouse IL‐6R*α* complex, salivaricin A2‐mouse IL‐21R complex, salivaricin B‐mouse IL‐6R*α* complex, salivaricin B‐mouse IL‐21R complex) all used AlphaFold (version 2.1.0) with the parameter “–model_preset = multimer” to predict their structures. Finally, the relaxed model was selected as the final prediction results and the surface state and contact interface of the above‐mentioned complexes were analyzed by PISA (version 1.52). Five mutants were constructed according to the results generated by PISA: salivaricin A2‐1 (mutation at the 2nd arginine residue), salivaricin A2‐2 (mutations at the 12th and 13th asparagine residues), salivaricin A2‐3 (mutations at 21st and 22nd cysteine residues), salivaricin B‐1 (mutation at the 23rd and 24th cysteine residue), and salivaricin B‐2 (mutations at the 11th glutamic acid and 13th arginine residues).

### CD Spectroscopy

CD experiments were performed at 25 °C using a Chirascan V100 (Jasco, Easton, MD, USA). Protein or peptide samples at 0.1–10 mg mL^−1^ were examined in a 1.0 mm‐path length Suprasil (quartz) cell (Hellma UK). CD spectra were collected over a wavelength range of 180–260 nm in 1 nm steps using 1s time per point with a bandwidth of 1 nm. All CD spectra were corrected for the baseline by subtracting the spectra of the corresponding IL‐6R or IL‐21R solutions and representing the average of three runs. The content of the secondary structures was estimated with the CDNN v2.1 software.^[^ ^40^ ^]^

### CIA Induction and Intervention

Male DBA/1 mice (6–8 weeks old) were purchased from Huafukang Co. Ltd. (Beijing, China) and fed under specific pathogen‐free conditions. All experiments were carried out in accordance with guidelines prescribed by the Animal Care and Use Committee of Peking University People's Hospital (2019PHE047). CIA induction was established by following a previously published protocol.^[^ ^41^ ^]^ Briefly, DBA/1 mice were immunized intradermally at the base of the tail with 200 µg of bovine type II collagen (CII, Chondrex) emulsified in complete Freund's adjuvant (Sigma‐Aldrich, St Louis, MO, USA) in equal volumes. Three weeks later, a booster was delivered using 100 µg CII emulsified in Freund's incomplete adjuvant. Mice were monitored for signs of arthritis after the booster immunization. Clinical score was assessed by using the following system as detailed previously:^[^ ^41^ ^]^ 0, normal; 1, erythema and swelling of one or several digits; 2, erythema and moderate swelling extending from the ankle to the mid‐foot (tarsals); 3, erythema and severe swelling extending from the ankle to the metatarsal joints; and 4, complete erythema and swelling encompassing the ankle, foot, and digits, resulting in deformity and/or ankyloses. The scores of all four limbs were summed, yielding total scores of 0–16 per mouse.

We randomized mice into control or treatment groups. Salivaricin A2 (50, 100, or 200 µg per mice) or B (50, 100, or 200 µg per mice) was supplemented intra‐orally, intraperitoneally or intragastrically three times a week. The preventive group started on day 1, and the therapeutic groups commenced after the onset of CIA (approximately day 26). At the study endpoints, mice were euthanized and serum samples were collected for cytokine and auto‐antibody detection. The spleens and joint DLNs (popliteal and axillary lymph nodes, DLN) were obtained from mice, sieved through a 70 µm cell strainer (Corning) in RPMI 1640 medium with 10% FBS and single‐cell suspensions (10^6^ cells per 100 µL) were prepared for flow cytometry. 10–23 mice were used in the indicated group, and data were pooled from two independent experiments.

### Radiography Evaluations and Histological Analyses

The paws from each mouse were collected and fixed in 4% paraformaldehyde (PFA) for 48 h, then scanned using a Micro‐CT scanner (Quantum FX, Caliper, USA). After that, the paws were decalcified in 5% EDTA, paraffin‐embedded, sectioned, and stained with hematoxylin and eosin. A microscopic assessment of sagittal sections was performed and histopathological changes were scored based on the following previously reported parameters:^[^ ^42^ ^]^ 0, normal synovium; 1, synovial membrane hypertrophy and cell infiltrates; 2, pannus and cartilage erosion; 3, major erosion of cartilage and subchondral bone; and 4, loss of joint integrity and ankylosis. The scores of all four limbs were summed and divided by 4, yielding average scores of 0–4 per mouse.

### Flow Cytometry

For intracellular cytokine analysis, cells were stimulated with 25 ng mL^−1^ PMA plus 1 µg mL^−1^ ionomycin for 4–5 h in the presence of 10 µg mL^−1^ brefeldin A (PeproTech). Cell surface markers were first stained, and the cells were then fixed and permeabilized with an intracellular staining buffer set (Thermo Fisher Scientific) following the manufacturer's protocol and stained with intracellular or intranuclear markers. Antibodies were purchased from BD Biosciences, eBiosciences (Thermo Fisher Scientific), or Biolegend. Murine Th1 cells were defined as CD4^+^IFN‐*γ* ^+^, Th17 cells as CD4^+^IL‐17A^+^, Treg cells as CD4^+^CD25^+^FOXP3^+^, Tfh cells as CD4^+^CD44^+^CXCR5^+^PD‐1^Hi^Bcl6^+^, GCB cells as B220^+^CD4^−^GL‐7^+^Fas^+^, and plasmablast as B220^+^CD4^−^CD138^+^. The human immune cell subsets were identified as follows: Treg were marked as CD3+CD4+CD25^hi^CD127^low^, pTfh as CD3^+^CD4^+^CD25^−^CD45RA^−^CXCR5^+^CCR7^low^PD1^+^, Th1 as CD3^+^CD4^+^ CD25^−^CD45RA^−^CCR7^low^CCR6^−^CXCR3^+^CCR4^−^, Th2 as CD3^+^CD4^+^ CD25^−^ CD45RA^−^CCR7^low^CCR6^−^CXCR3^−^CCR4^+^, Th17 as CD3^+^CD4^+^CD25^−^CD45RA^−^ CCR7^low^CCR6^+^CXCR3^−^CCR4^+^, plasma cells as CD3^−^CD19^+^CD20^−^CD38^+^CD27^+^, DC cells as Lineage‐HLA‐DR^+^, pDC cells as Lin‐HLA‐DR^+^CD123^hi^CD11C^−/dim^, mDC cells as Lineage‐HLA‐DR^+^CD123^dim^CD11C^+^, CD56^hi^NK cells as CD3^−^CD56^hi^CD16^−/dim^, CD56^dim^NK cells as CD3^−^CD56^dim^CD16^+^, NKT cells as CD3^+^CD56^+^. Please see Figures S11–S13, Supporting Information, for the detailed gating strategies. Flow cytometry was performed using FACSAriaII (BD Biosciences) and the data was analyzed using FlowJo v10.0.7 software (Tree Star Inc.).

### Cytokine and Auto‐Antibody Detection

The concentrations of cytokines (IL‐21, IL‐17A, IL‐6, IL‐10, TNF‐*α*, or IFN‐*γ*) were measured using ELISA kits (Multisciences), and the titer of collagen‐specific antibody was analyzed using a mouse anti‐bovine type II collagen IgG antibody assay kit (Chondrex). All measurements followed kit protocols as described in the manufacturer's instructions.

### Statistical Analyses

The differentially expressed lantibiotics were identified based on the Wilcoxon rank‐sum test. *P*‐value < 0.05 was considered statistically significant. Correlation analysis was carried out using Spearman's rank correlation statistical measurement system.^[^ ^43^ ^]^ The difference in cytokines, antibodies, lymphocytes, and RNA abundance were analyzed by one‐way ANOVA followed by Holm‐Sidak's multiple comparisons tests or Mann‐Whitney nonparametric test, and the differences in arthritis scores and incidence between groups were determined by two‐way ANOVA followed by Tukey's multiple comparisons test and Kaplan‐Meier analysis with log‐rank test, respectively. The statistical analyses were performed by the GraphPad prism v7.00 software (GraphPad, San Diego, CA, USA). A two‐sided *P*‐value of < 0.05 was considered statistically significant.

## Conflict of Interest

The authors declare no conflict of interest.

## Author Contributions

J.L., J.J., and S.L. contributed equally to this work. Z.G.L., J.H., J.W., and J.J.Q. conceived and designed the project. J.L., J.Y.J., F.H, Y.Z., H.Y., R.L., and Y.S.Z. were in charge of participant enrollment and sample collection. Q.L.Y., X.Z., X.H.X., and H.H.Y. carried out DNA extraction, PCR, and microbiota sequencing. S.H.L., R.C.G., B.B. X., and J.W. completed all the bioinformatic and statistical analyses of the microbiome. J.L., J.Y.J., Y.H.Z., Y.Z., W.J.X., Y.B.J., F.H., X.H.X., L.S., and B.B.X. performed all the in vitro and in vivo experiments. J.L., S.H.L., J.Y.J., R.C.G., X.L.S., J.P.G., and F.L.H., and conducted data acquiring and processing as well as figure preparations. J.L., SH.L., and J.Y.J. wrote the manuscript with input and edits from Z.G.L., J.W., J.H., H.Q., X.H.G., Y.D.L., T.P.L., and L.J.W. All the authors have revised and approved the manuscript submission.

## Supporting information

Supporting Information

Supporting Information

## Data Availability

The data that support the findings of this study are openly available in [China National GeneBank] at [https://db.cngb.org], reference numbers [2433] and [1841].

## References

[1] a) W. E. Ruff , T. M. Greiling , M. A. Kriegel , Nat. Rev. Microbiol. 2020, 18, 521; 32457482 10.1038/s41579-020-0367-2

[2] A. N. Skelly , Y. Sato , S. Kearney , K. Honda , Nat. Rev. Immunol. 2019, 19, 305. 30858494 10.1038/s41577-019-0144-5

[3] M. M. Zaiss , H. J. Joyce Wu , D. Mauro , G. Schett , F. Ciccia , Nat. Rev. Rheumatol. 2021, 17, 224. 33674813 10.1038/s41584-021-00585-3

[4] J. S. Smolen , D. Aletaha , A. Barton , G. R. Burmester , P. Emery , G. S. Firestein , A. Kavanaugh , I. B. McInnes , D. H. Solomon , V. Strand , K. Yamamoto , Nat. Rev. Dis. Primers 2018, 4, 18001. 29417936 10.1038/nrdp.2018.1

[5] a) S. Abdollahi‐Roodsaz , L. A. Joosten , M. I. Koenders , I. Devesa , M. F. Roelofs , T. R. Radstake , M. Heuvelmans‐Jacobs , S. Akira , M. J. Nicklin , F. Ribeiro‐Dias , W. B. van den , Berg , J. Clin. Invest. 2008, 118, 205; 18060042 10.1172/JCI32639 PMC2104479

[6] a) J. U. Scher , C. Ubeda , M. Equinda , R. Khanin , Y. Buischi , A. Viale , L. Lipuma , M. Attur , M. H. Pillinger , G. Weissmann , D. R. Littman , E. G. Pamer , W. A. Bretz , S. B. Abramson , Arthritis Rheum. 2012, 64, 3083; 22576262 10.1002/art.34539 PMC3428472

[7] a) K. A. Krautkramer , J. Fan , F. Backhed , Nat. Rev. Microbiol. 2021, 19, 77; 32968241 10.1038/s41579-020-0438-4

[8] W. Li , S. Hang , Y. Fang , S. Bae , Y. Zhang , M. Zhang , G. Wang , M. D. McCurry , M. Bae , D. Paik , E. A. Franzosa , F. Rastinejad , C. Huttenhower , L. Yao , A. S. Devlin , J. R. Huh , Cell Host Microbe 2021, 29, 1366. 34416161 10.1016/j.chom.2021.07.013 PMC9064000

[9] J. Li , S. Li , J. Jin , R. Guo , X. Sun , J. Guo , F. Hu , Y. Liu , Y. Jin , Y. Zhou , W. Xiao , Y. Zhong , F. Huang , H. Pan , R. Yang , Y. Zhou , K. Deng , L. Wu , L. Liu , J. Qin , J. Wang , J. He , Z. Li , BioRxiv 2019. https://www.biorxiv.org/content/10.1101/719807v1.

[10] a) A. Barbour , P. Wescombe , L. Smith , Trends Microbiol. 2020, 28, 578; 32544444 10.1016/j.tim.2020.03.001

[11] R. E. Hancock , E. F. Haney , E. E. Gill , Nat. Rev. Immunol. 2016, 16, 321. 27087664 10.1038/nri.2016.29

[12] J. Kindrachuk , H. Jenssen , M. Elliott , A. Nijnik , L. Magrangeas‐Janot , M. Pasupuleti , L. Thorson , S. Ma , D. M. Easton , M. Bains , B. Finlay , E. J. Breukink , H. Georg‐Sahl , R. E. Hancock , Innate Immun. 2013, 19, 315. 23109507 10.1177/1753425912461456

[13] H. Grasemann , F. Stehling , H. Brunar , R. Widmann , T. W. Laliberte , L. Molina , G. Doring , F. Ratjen , Chest 2007, 131, 1461. 17494794 10.1378/chest.06-2085

[14] G. Wang , X. Li , Z. Wang , Nucleic Acids Res. 2016, 44, D1087. 26602694 10.1093/nar/gkv1278 PMC4702905

[15] a) O. Hyink , P. A. Wescombe , M. Upton , N. Ragland , J. P. Burton , J. R. Tagg , Appl. Environ. Microbiol. 2007, 73, 1107; 17194838 10.1128/AEM.02265-06 PMC1828679

[16] A. Barbour , J. Tagg , O. K. Abou‐Zied , K. Philip , Sci. Rep. 2016, 6, 31749. 27526944 10.1038/srep31749 PMC4985645

[17] G. V. Walker , N. C. K. Heng , A. Carne , J. R. Tagg , P. A. Wescombe , Microbiology 2016, 162, 476. 26744310 10.1099/mic.0.000237

[18] P. A. Wescombe , K. H. Dyet , K. P. Dierksen , D. A. Power , R. W. Jack , J. P. Burton , M. A. Inglis , A. L. Wescombe , J. R. Tagg , Internet J. Microbiol. 2012, 2012, 738503. 10.1155/2012/738503 PMC3332205 22567013

[19] K. Vaillancourt , G. LeBel , M. Frenette , N. Fittipaldi , M. Gottschalk , D. Grenier , PLoS One 2015, 10, e0145854. 26709705 10.1371/journal.pone.0145854 PMC4692507

[20] a) J. Deng , Y. Wei , V. R. Fonseca , L. Graca , D. Yu , Nat. Rev. Rheumatol. 2019, 15, 475; 31289377 10.1038/s41584-019-0254-2

[21] a) R. Spolski , W. J. Leonard , Nat. Rev. Drug Discovery 2014, 13, 379; 24751819 10.1038/nrd4296

[22] a) X. Liu , X. Chen , B. Zhong , A. Wang , X. Wang , F. Chu , R. I. Nurieva , X. Yan , P. Chen , L. G. van der Flier , H. Nakatsukasa , S. S. Neelapu , W. Chen , H. Clevers , Q. Tian , H. Qi , L. Wei , C. Dong , Nature 2014, 507, 513; 24463518 10.1038/nature12910 PMC4012617

[23] a) R. I. Nurieva , Y. Chung , G. J. Martinez , X. O. Yang , S. Tanaka , T. D. Matskevitch , Y. H. Wang , C. Dong , Science 2009, 325, 1001; 19628815 10.1126/science.1176676 PMC2857334

[24] C. Garbers , S. Heink , T. Korn , S. Rose‐John , Nat. Rev. Drug Discovery 2018, 17, 395. 29725131 10.1038/nrd.2018.45

[25] S. Crotty , Immunity 2019, 50, 1132. 31117010 10.1016/j.immuni.2019.04.011 PMC6532429

[26] a) Y. S. Choi , D. Eto , J. A. Yang , C. Lao , S. Crotty , J. Immunol. 2013, 190, 3049; 23447690 10.4049/jimmunol.1203032 PMC3626564

[27] A. Vogelzang , H. M. McGuire , D. Yu , J. Sprent , C. R. Mackay , C. King , Immunity 2008, 29, 127. 18602282 10.1016/j.immuni.2008.06.001

[28] R. Nurieva , X. O. Yang , G. Martinez , Y. Zhang , A. D. Panopoulos , L. Ma , K. Schluns , Q. Tian , S. S. Watowich , A. M. Jetten , C. Dong , Nature 2007, 448, 480. 17581589 10.1038/nature05969

[29] P. Dinesh , M. Rasool , J. Cell. Physiol. 2018, 233, 3918. 28833093 10.1002/jcp.26158

[30] a) V. M. Holers , M. K. Demoruelle , K. A. Kuhn , J. H. Buckner , W. H. Robinson , Y. Okamoto , J. M. Norris , K. D. Deane , Nat. Rev. Rheumatol. 2018, 14, 542; 30111803 10.1038/s41584-018-0070-0 PMC6704378

[31] J. J. Johnston , R. Douglas , Postgrad. Med. J. 2018, 94, 398. 29884749 10.1136/postgradmedj-2018-135602

[32] H. Nave , A. Gebert , R. Pabst , Anat. Embryol. 2001, 204, 367. 10.1007/s004290100210 11789984

[33] a) C. Jorgensen , I. Couret , F. Canovas , C. Bologna , J. Brochier , T. Reme , J. Sany , Autoimmunity 1996, 24, 179; 9020410 10.3109/08916939608995363

[34] P. A. Wescombe , M. Upton , K. P. Dierksen , N. L. Ragland , S. Sivabalan , R. E. Wirawan , M. A. Inglis , C. J. Moore , G. V. Walker , C. N. Chilcott , H. F. Jenkinson , J. R. Tagg , Appl. Environ. Microbiol. 2006, 72, 1459. 16461700 10.1128/AEM.72.2.1459-1466.2006 PMC1392966

[35] P. A. Wescombe , J. P. Burton , P. A. Cadieux , N. A. Klesse , O. Hyink , N. C. Heng , C. N. Chilcott , G. Reid , J. R. Tagg , Antonie Van Leeuwenhoek 2006, 90, 269. 16871420 10.1007/s10482-006-9081-y

[36] a) G. Karthikeyan , L. Guilherme , Lancet 2018, 392, 161; 30025809 10.1016/S0140-6736(18)30999-1

[37] D. Aletaha , T. Neogi , A. J. Silman , J. Funovits , D. T. Felson , C. O. Bingham, 3rd , N. S. Birnbaum , G. R. Burmester , V. P. Bykerk , M. D. Cohen , B. Combe , K. H. Costenbader , M. Dougados , P. Emery , G. Ferraccioli , J. M. Hazes , K. Hobbs , T. W. Huizinga , A. Kavanaugh , J. Kay , T. K. Kvien , T. Laing , P. Mease , H. A. Menard , L. W. Moreland , R. L. Naden , T. Pincus , J. S. Smolen , E. Stanislawska‐Biernat , D. Symmons , et al., Arthritis Rheum. 2010, 62, 2569. 20872595 10.1002/art.27584

[38] J. Sun , R. Li , J. Guo , Y. Jia , X. Sun , Y. Liu , Y. Li , F. Huang , L. Lu , Z. Li , Arthritis Rheum. 2012, 64, 2158. 22231228 10.1002/art.34372

[39] Q. Wang , M. V. Liberti , P. Liu , X. Deng , Y. Liu , J. W. Locasale , L. Lai , Cell Chem. Biol. 2017, 24, 55. 28042046 10.1016/j.chembiol.2016.11.013 PMC5915676

[40] a) A. Micsonai , F. Wien , L. Kernya , Y. H. Lee , Y. Goto , M. Refregiers , J. Kardos , Proc. Natl. Acad. Sci. USA 2015, 112, E3095; 26038575 10.1073/pnas.1500851112 PMC4475991

[41] D. D. Brand , K. A. Latham , E. F. Rosloniec , Nat. Protoc. 2007, 2, 1269. 17546023 10.1038/nprot.2007.173

[42] M. Nishikawa , A. Myoui , T. Tomita , K. Takahi , A. Nampei , H. Yoshikawa , Arthritis Rheum. 2003, 48, 2670. 13130488 10.1002/art.11227

[43] M. A. Zapala , N. J. Schork , Proc. Natl. Acad. Sci. USA 2006, 103, 19430. 17146048 10.1073/pnas.0609333103 PMC1748243

