# Supplementary material for: Tonsillar Microbiome‐Derived Lantibiotics Induce Structural Changes of IL‐6 and IL‐21 Receptors and Modulate Host Immunity
Source: Adv Sci (Weinh). 2022 Aug 28;9(30):2202706. doi: 10.1002/advs.202202706 (PMC9596850; doi:10.1002/advs.202202706)
Supplement: Supplementary file 1 — Supporting Information [file ADVS-9-2202706-s002.pdf]

ADVANCED SCIENCE
Open Access

Supporting Information

for *Adv. Sci.*, DOI 10.1002/advs.202202706

Tonsillar Microbiome-Derived Lantibiotics Induce Structural Changes of IL-6 and IL-21 Receptors and Modulate Host Immunity

Jing Li, Jiayang Jin, Shenghui Li, Yan Zhong, Yuebo Jin, Xuan Zhang, Binbin Xia, Yinhua Zhu, Ruochun Guo, Xiaolin Sun, Jianping Guo, Fanlei Hu, Wenjing Xiao, Fei Huang, Hua Ye, Ru Li, Yunshan Zhou, Xiaohong Xiang, Haihong Yao, Qiulong Yan, Li Su, Lijun Wu, Tuoping Luo, Yudong Liu, Xiaohuan Guo, Junjie Qin, Hai Qi, Jing He, Jun Wang* and Zhanguo Li**

Supporting Information for

Tonsillar microbiome-derived lantibiotics induce structural changes of IL-6 and IL-21 receptors and modulate host immunity

Jing Li[1,2†], *Jiayang Jin*[1,2†], *Shenghui Li*[3,4†], *Yan Zhong*[1,2,5], *Yuebo Jin*[1,2], *Xuan Zhang*[6,7], *Binbin Xia*[6,7], *Yinhua Zhu*[8,9], *Ruochun Guo*[4], *Xiaolin Sun*[1,2], *Jianping Guo*[1,2], *Fanlei Hu*[1,2], *Wenjing Xiao*[1,2,10], *Fei Huang*[1,2], *Hua Ye*[1,2], *Ru Li*[1,2], *Yunshan Zhou*[1,2], *Xiaohong Xiang*[1,2], *Haihong Yao*[1,2], *Qiulong Yan*[11], *Li Su*[12], *Lijun Wu*[5], *Tuoping Luo*[8,9], *Yudong Liu*[13], *Xiaohuan Guo*[14], *Junjie Qin*[4], *Hai Qi*[15], *Jing He*[1,2]*, *Jun Wang*[6,7]*, *Zhanguo Li*[1,2,16]*

Department of Rheumatology and Immunology, Peking University People's Hospital, Beijing, 100044, China

[2]Beijing Key Laboratory for Rheumatism Mechanism and Immune Diagnosis (BZ0135), Beijing, 100044, China

[3]Key Laboratory of Precision Nutrition and Food Quality, Department of Nutrition and Health, China Agricultural University, Beijing, 100083, China

[4]Promegene Translational Research Institute, Shenzhen, 518110, China

[5]Department of Rheumatology and Immunology, People's Hospital of Xin Jiang Uygur Autonomous Region, Urumqi, 830001, China

[6]CAS Key Laboratory for Pathogenic Microbiology and Immunology, Institute of Microbiology, Chinese Academy of Sciences, Beijing, 100101, China

[7]University of Chinese Academy of Sciences, Beijing, 100049, China

[8]Peking-Tsinghua Center for Life Sciences, Academy for Advanced Interdisciplinary Studies, Peking University, Beijing, 100871, China

[9]Key Laboratory of Bioorganic Chemistry and Molecular Engineering, Ministry of Education and Beijing National Laboratory for Molecular Science, College of Chemistry and Molecular Engineering, Peking University, Beijing, 100871, China

[10]Emergency Department, Peking University People's Hospital, Beijing, 100044, China

[11]Department of Microbiology, College of Basic Medical Sciences, Dalian Medical

University, Dalian, 116044, China

[12]Center of Medical and Health Analysis, Peking University, Beijing, 100191, China

[13]Department of Clinical Laboratory, Peking University People's Hospital, Beijing, 100044, China

[14]Institute for Immunology, School of Medicine, Tsinghua University, Beijing 100084, China

[15]Tsinghua-Peking Center for Life Sciences, Tsinghua University, Beijing, 100084, China

[16]State Key Laboratory of Natural and Biomimetic Drugs, School of Pharmaceutical Sciences, Peking University, Beijing, 100191, China

*** Correspondence**

Professor Zhanguo Li, Email: li99@bjmu.edu.cn;

Professor Jun Wang, Email: junwang@im.ac.cn;

Professor Jing He, Email: hejing1105@126.com.

[†]These authors contributed equally to this work.

Supplementary Figure Legends

Figure S1

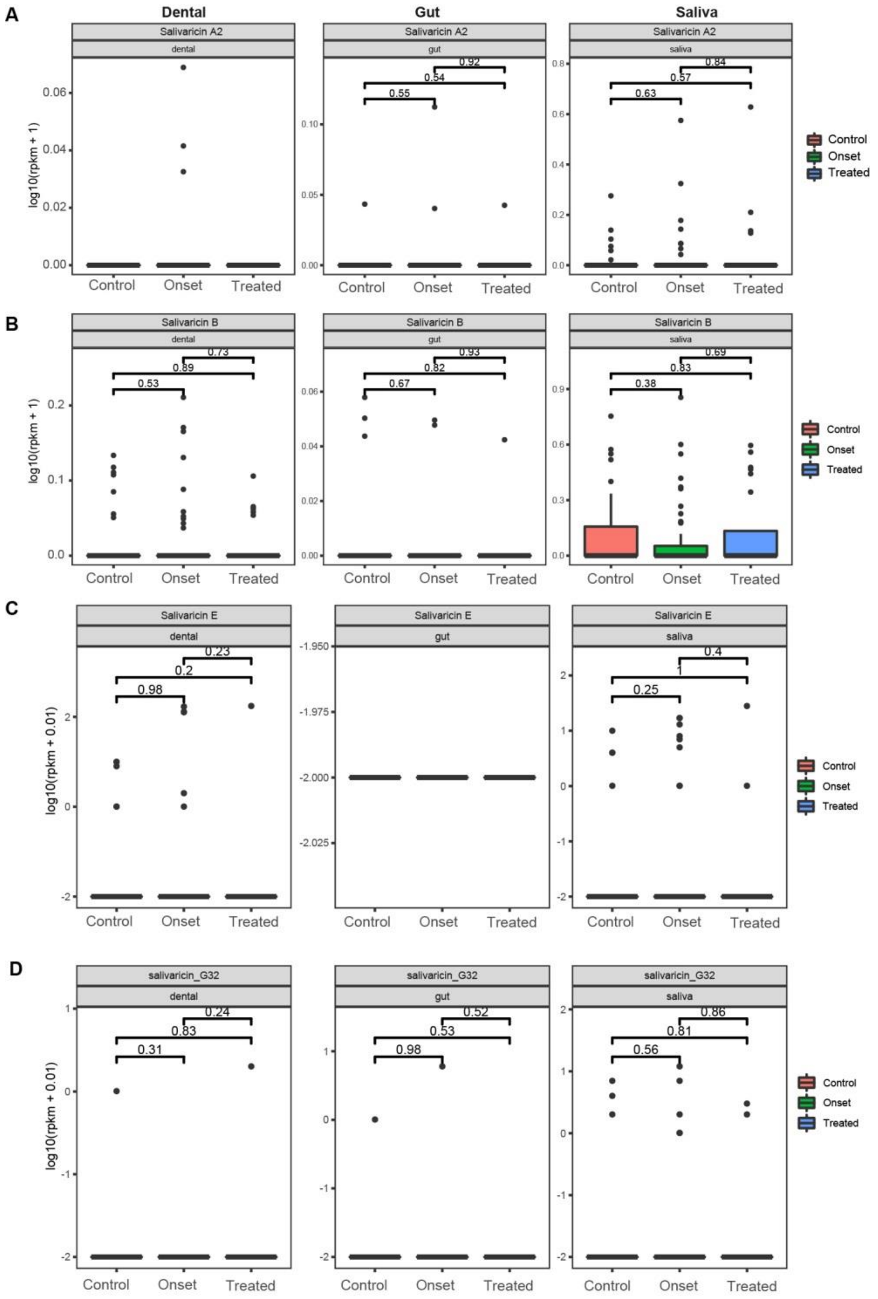

Figure S1. The expressions of lantibiotic-encoding genes in the oral and gut microbiomes. A-D) Boxplot showing the relative abundances of lantibiotic-encoding genes (salivaricin A2, B, E, and G32) in the saliva, dental plaque and gut microbiomes of rheumatoid arthritis (RA) patients and healthy controls (Zhang et al., 2015). Boxes represented the interquartile range between the first and third quartiles and median (internal line). Whiskers denoted the lowest and highest values within 1.5 times the range of the first and third quartiles, respectively; and dots represented outlier samples beyond the whiskers. RPKM, reads per kilobase per million mapped reads. Wilcoxon rank sum test.

Figure S2

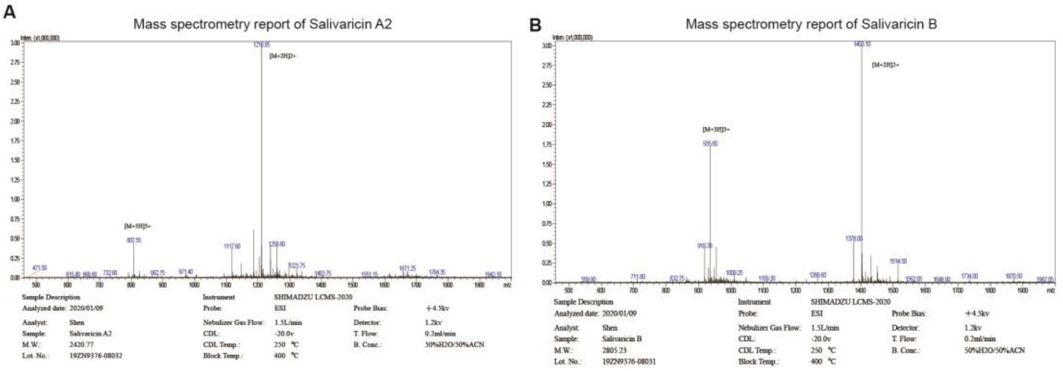

**Product Name:**	Peptide 2 : Salivaricin A2
**Lot Num:**	19ZN9376-08032
**Sequence:**	KRGTGWFATITDDCPNSVFVCC
**Molecular Weight**	2420.77
**Mass Spectral Analysis:**	see attached MS spectrogram
**HPLC Analysis:**	Peptide purity: 98.89%
**Column :**	agilent ZORBAX 300SB-C18 5um 4.6*250mm
**Mobile phase:**	A:0.1%TFA in 100%H2O<br>B:ACN
**Appearance:**	White lyophilized powder
**Counter Ion:**	Trifluoroacetate
Endotoxin Unit	3.6 EU/mg (< 5EU/mg)

**Product Name:**	Peptide 1 : Salivaricin B
**Lot Num:**	19ZN9376-08031
**Sequence:**	GGGVIQTISHECRMNSWQFLFTCCS
**Molecular Weight**	2805.23
**Mass Spectral Analysis:**	see attached MS spectrogram
**HPLC Analysis:**	Peptide purity: 98.73%
**Column :**	agilent ZORBAX 300SB-C18 5um 4.6*250mm
**Mobile phase:**	A:0.1%TFA in 100%H2O<br>B:ACN
**Appearance:**	White lyophilized powder
**Counter Ion:**	Trifluoroacetate
Endotoxin Unit	2.8 EU/mg (< 5EU/mg)

Figure S2. Mass spectrometry reports and endotoxin examining results of chemosynthetic salivaricin A2 and B. A and B) Salivaricin A2 (A, KRGTGWFATITDDCPNSVFVCC) and salivaricin B (B, GGGVIQTISHECRMNSWQFLFTCCS) were synthesized, and then were purified by high-performance liquid chromatography with a purity of more than 96% and identified by Liquid chromatography-mass spectrometer.

Figure S3

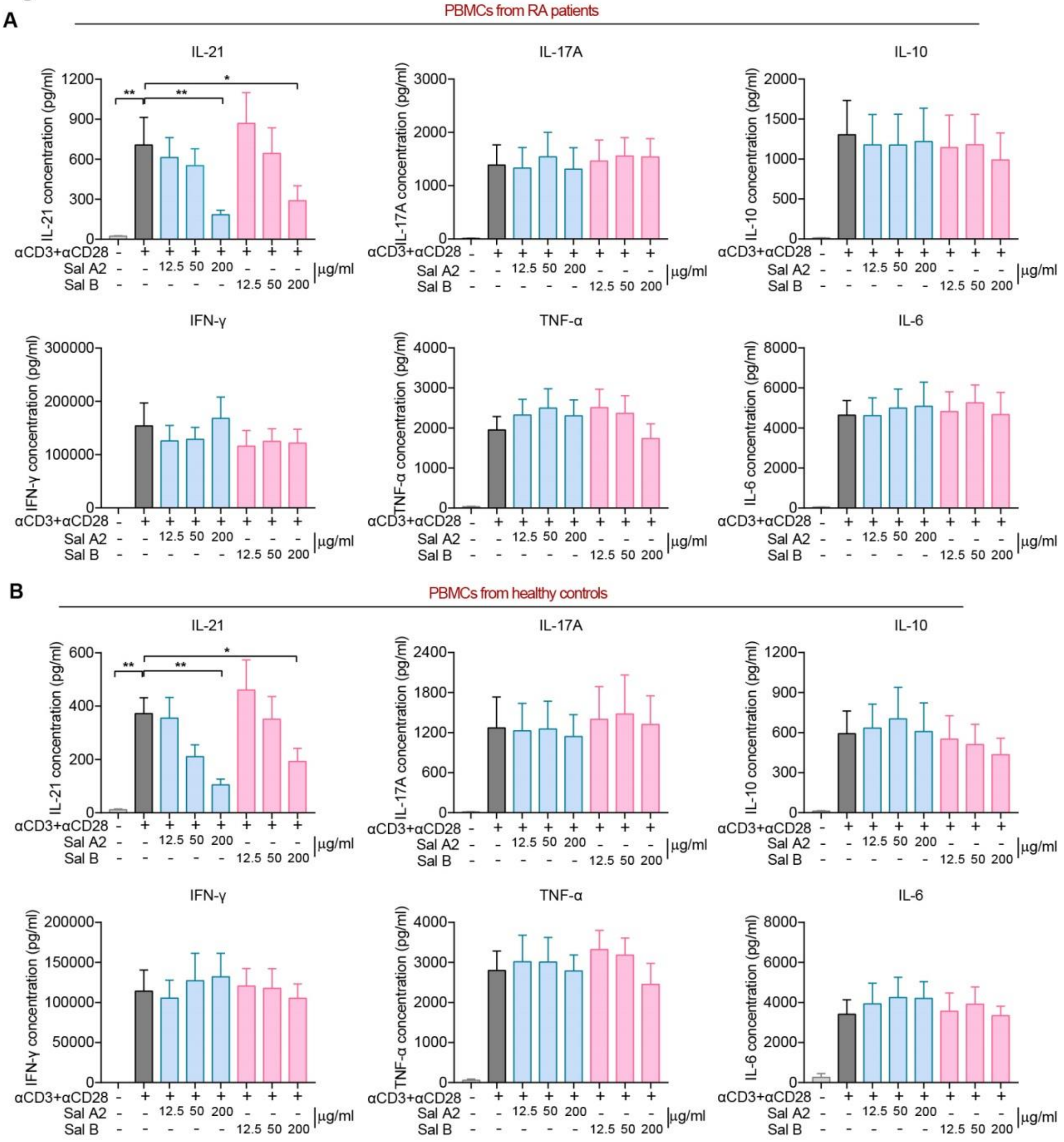

Figure S3. Salivaricins down-regulate IL-21 production in human PBMCs. A and B) Peripheral blood mononuclear cells (PBMCs) were isolated from RA patients (A, n=7-11) and healthy individuals (B, n=6-8), and followed by culturing under αCD3 and αCD28 antibodies activation with or without salivaricins co-incubation for 3 days. Levels of interleukin (IL) -21, IL-17A, IL-10, IFN-γ, TNF-α, and IL-6 in the conditional medium were measured by ELISA kits. SalA2: salivaricin A2; SalB: salivaricin B. Data are expressed as the mean ± sem. Significance determined using one-way ANOVA followed by Holm-Sidak's multiple comparisons test. *$P < 0.05$, **$P < 0.01$.

Figure S4

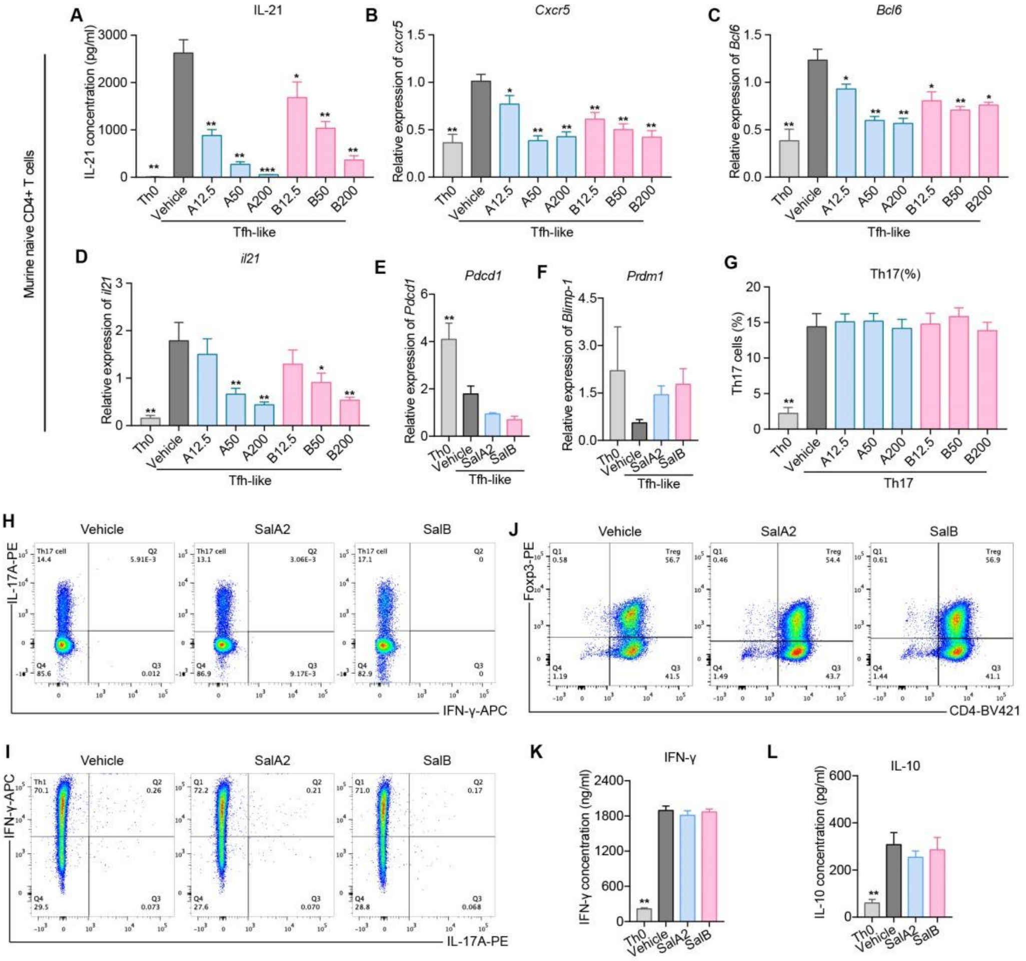

Figure S4. Salivaricins inhibit Tfh cell differentiation and IL-21 production *in vitro*. A) IL-21 concentrations were tested by ELISA kits in Tfh-like cells. n=10. **B-F)** Expressions of genes (*Cxcr5*, *Bcl6*, *il21*, *Pdcd1*, *and Prdm1*) measured by qPCR in Tfh-like cells. n=6-10**. G)** The ratio of Th17 cells was detected by flow cytometry. n=6-9. **H-J)** Representative flow cytometry graphs showing frequencies of Th17 (H, $CD4^{+}IL\text{-}17A^{+}IFN\text{-}\gamma^{-}$), Th1 (I, $CD4^{+}IL\text{-}17A^{-}IFN\text{-}\gamma+$) and iTreg (J, $CD4^{+}Foxp3^{+}$) cells. **K and L)** IFN-γ and IL-10 concentrations were tested by ELISA kits in Th1 (K, n=9) and iTreg (L, n=4-8) cells, respectively. Data are expressed as the mean ± sem. Significance was determined using one-way ANOVA followed by Holm-Sidak's multiple comparisons test (compared with the vehicle group). *$P < 0.05$, **$P < 0.01$.

Figure S5

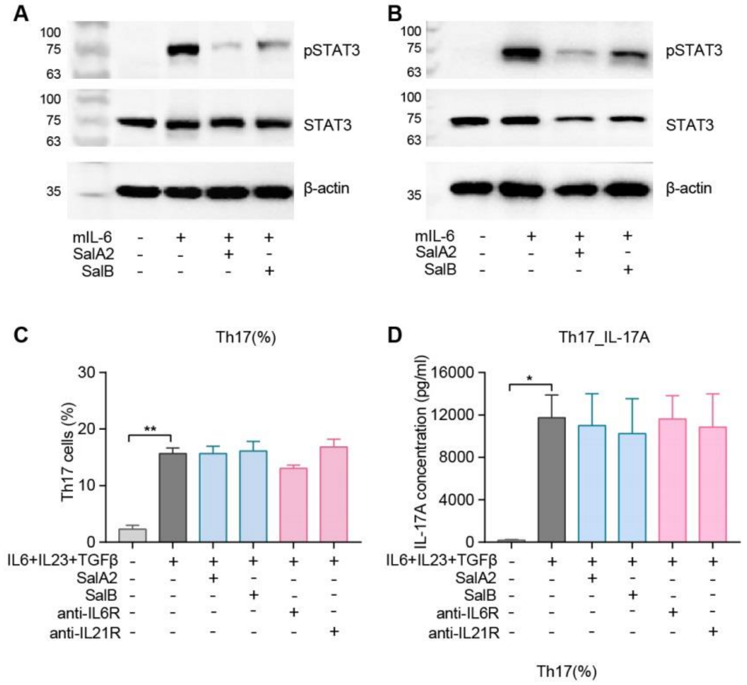

Figure S5. Salivaricins inhibit the IL-6/IL-21-STAT3 signaling pathway. A,B) Assessment of phosphorylation level of STAT3 by western blot in Tfh-like cells with or without salivaricin-treatment. n=3. C,D) Assessment of the proportion of Th17 cells using flow cytometry (C) and IL-17A level by ELISA (D) in the supernatant of Th17 cells. n=7-9. Data are expressed as the mean ± sem. Significance was assessed using one-way ANOVA followed by Holm-Sidak's multiple comparisons tests (C,D). $^*P < 0.05$, $^{**}P < 0.01$.

Figure S6

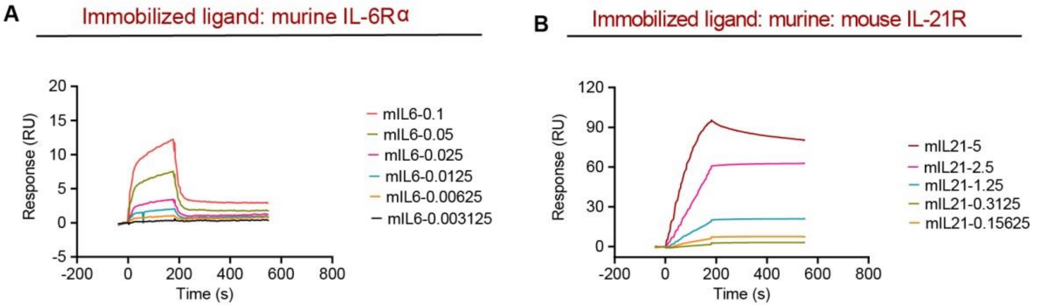

Figure S6. The binding affinities of IL-6 and IL-21 to their receptors. A) Surface plasmon resonance (SPR) sensorgram for IL-6 binding to the immobilized murine IL-6Rα. IL-6Rα: IL-6 receptor subunits alpha (20nM); mIL-6: murine IL-6 (0.003125-0.1 μM). **B)** SPR sensorgram for IL-21 binding to the immobilized murine IL-21R . IL-21R: IL-21 receptor (20nM); mIL-21: murine IL-21 (0.625-5 nM).

Figure S7

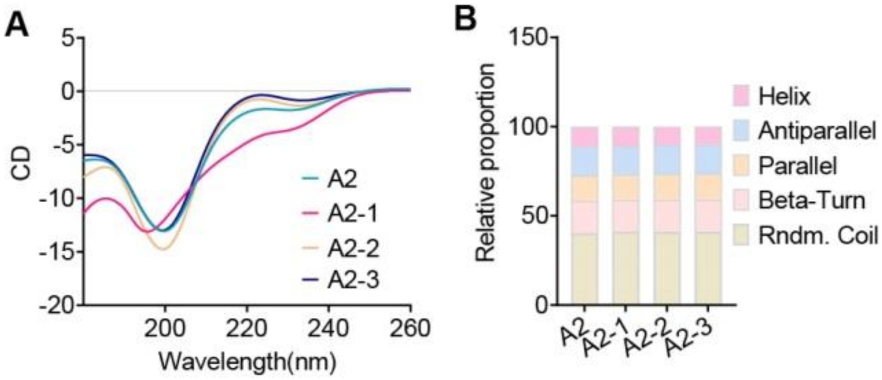

Figure S7. The conformation of salivaricin A2 and its mutants. The circular dichroism (CD) spectrum and the statistical proportions of different secondary structures (e.g., alpha-helix, beta-barrels and random coils) of salivaricin A2 and its mutants. A2: salivaricin A2; A2-2: salivaricin A2 with mutation at the 2nd arginine residue; A2-2: salivaricin A2 with mutations at the 12nd and 13th asparagine residues; A2-2: salivaricin A2 with mutations at 21st and 22nd cysteine residues.

Figure S8

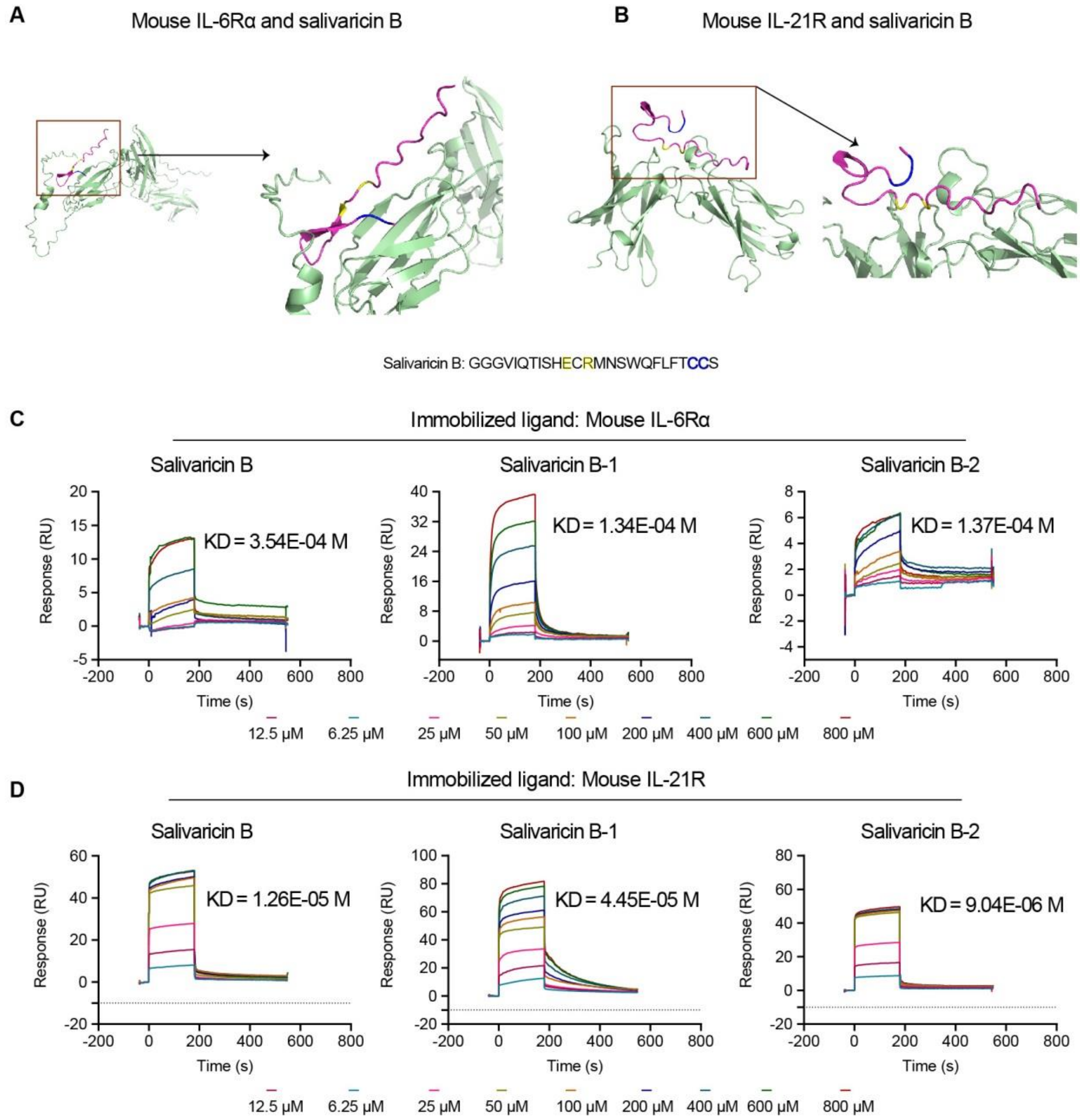

Figure S8. Identification of binding sites of salivaricins B to the receptors. A,B) Computational approach was used to predict the potential amino acid sites of salivaricin B binding to mouse IL-6Rα and IL-21R, identifying four residues according to the results generated by PISA for further validation. **C)** SPR sensorgram for the bindings of salivaricin B mutants (12.5-800 μM) to the immobilized murine IL-6R (20 nM), identifying KD values of 354 μM for salivaricin B, 134 μM for salivaricin B-1 (mutation at the 23th and 24th cysteine residue), 137 μM for salivaricin B-2 (mutations at the 11st glutamic acid and 13th arginine residues) with murine IL-6Rα. **D)** SPR sensorgram for the bindings of salivaricin B mutants (12.5-800 μM) to the immobilized murine IL-21R (20 nM), identifying KD values of 12.6 μM for salivaricin B, 44.5 μM for salivaricin B-1, and 9.04 μM for salivaricin B-2 with murine IL-21R.

Figure S9

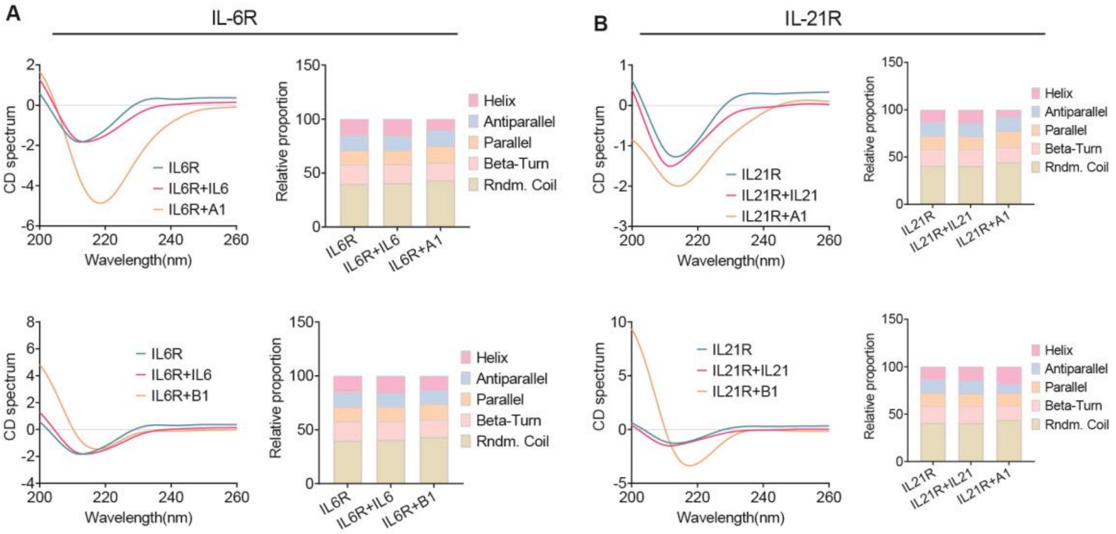

Figure S9. Salivaricins induce conformational changes of IL-6 and IL-21 receptors. A) The circular dichroism (CD) spectrum and the statistical proportions of different secondary structures (e.g., alpha-helix, beta-barrels and random coils) of IL-6Rα in the presence or absence of IL-6 (A) and salivaricins. **B)** The CD spectrum and the statistical proportions of different secondary structures of IL-21R in the presence or absence of IL-21 and salivaricins. mIL-6R: 0.1 mg/ml; SalA2: 1 mg/ml; SalB: 1 mg/ml; mIL-6: 0.1 mg/ml. mIL-21R: 0.1 mg/ml; SalA2: 1 mg/ml; SalB: 1 mg/ml; mIL-21: 0.1 mg/ml.

Figure S10

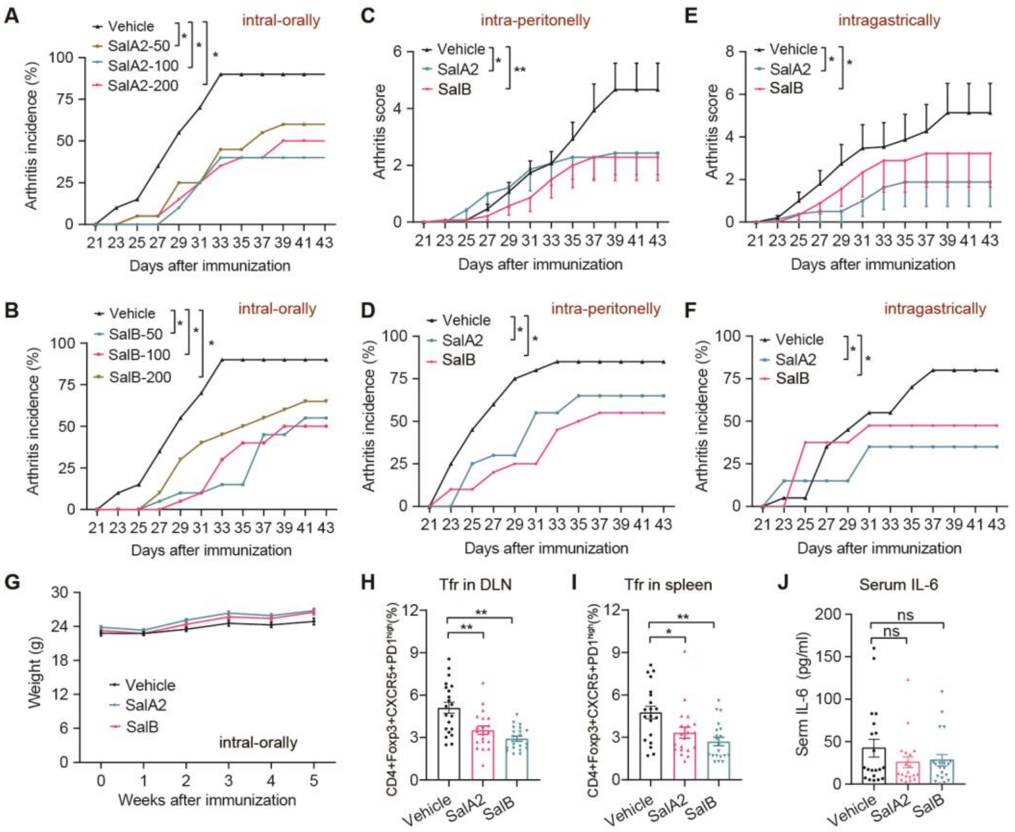

Figure S10. Salivaricins protect against experimental arthritis in mice. A and B) Clinical arthritis incidences in collagen-induced arthritis (CIA) mice with or without administrations of salivaricin A2 or B intral-orally. n=20 in each group, SalA2: salivaricin A2 (50, 100, or 200 μg/mice); SalB: salivaricin B (50, 100, or 200 μg/mice). **C and D)** Clinical arthritis scores (C) and incidences (D) in CIA mice with or without administrations of salivaricin A2 or B intra-peritonelly. n=14 in each group, SalA2=100 μg/mice; SalB=100 μg/mice. **E and F)** Clinical arthritis scores (E) and incidences (F) in CIA mice with or without administrations of salivaricin A2 or B intragastrically. n=15 in vehicle, n=8 in SalA2 (100 μg/mice), n=9 in SalB (100 μg/mice). **G)** Body weight of CIA mice in the indicated groups. n=20/group. **H and I)** Graphs showing frequencies of Tfr cells ($CD4^+Foxp3^+CXCR5^+PD1^{hi}$) in the DLNs and spleens of the indicated groups. n=20/group. **J)** Serum concentrations of IL-6. For G-J, n=20/group, SalA2=100 μg/mice and SalB=100μg/mice treated by intraorally. Data were pooled from two independent experiments and expressed as mean ± sem. Significance determined using two-way ANOVA followed by Tukey's multiple comparisons test (A, C, E, G), Kaplan-Meier analysis with log-rank test (B, D, and F), or Mann-Whitney test (H-J), **P* < 0.05, ***P* < 0.01. ns: not-significant.

Figure S11

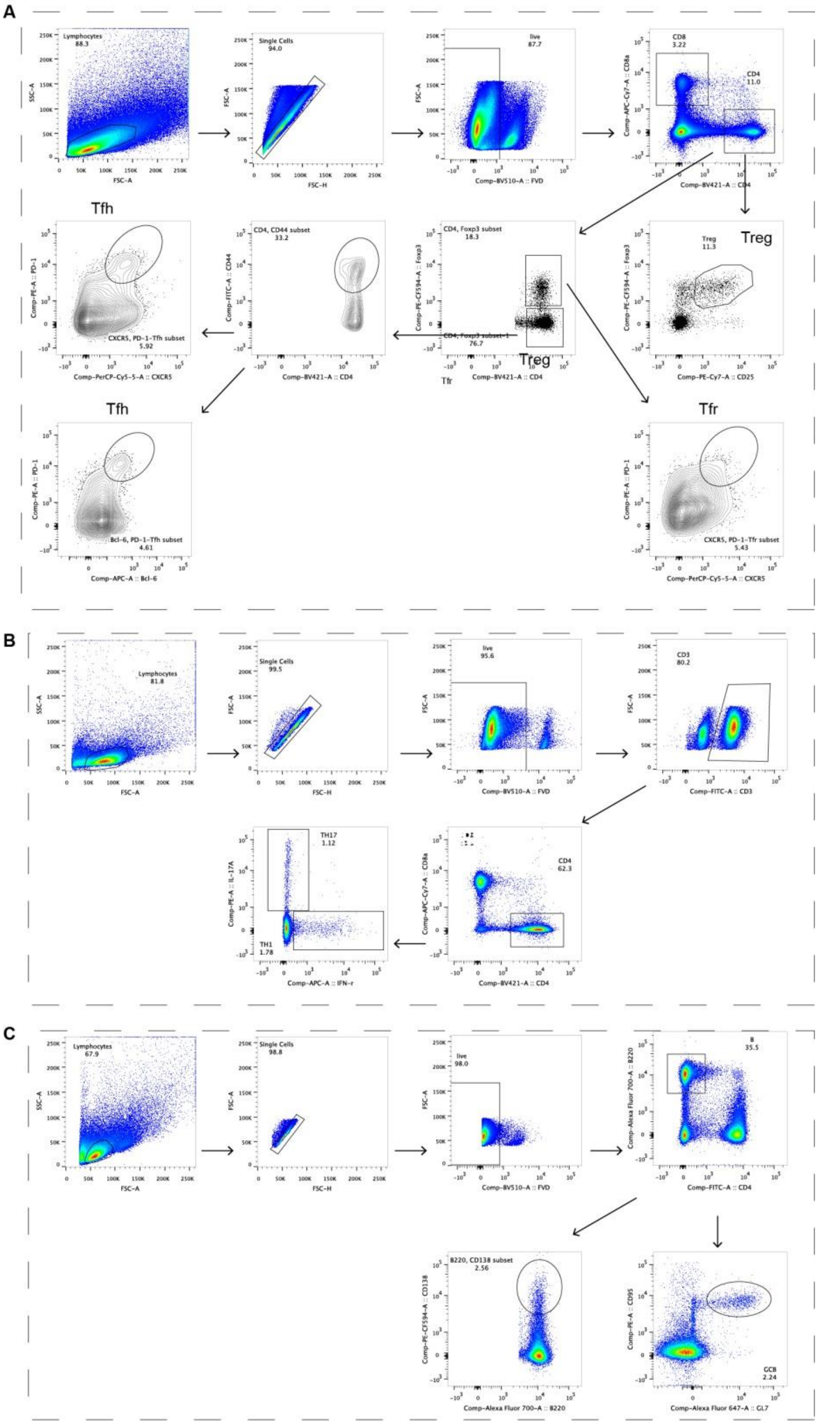

Figure S11. Schematic diagram displaying the gating strategies for lymphocyte subsets in mice. A**)** Gating strategies for Tfh and Treg cells. Murine Treg cells were defined as CD4$^+$CD25$^+$FOXP3$^+$, Tfh cells as CD4$^+$ Foxp3$^-$CD44$^+$CXCR5$^+$PD-1^{hi}Bcl6$^+$, and Tfr cells as CD4$^+$Foxp3$^+$CXCR5$^+$PD-1^{hi}. B) Gating strategies for Th1 and Th17 cells. Murine Th1 cells were defined as CD4$^+$IFN-γ^+, and Th17 cells as CD4$^+$IL-17A$^+$. C) Gating strategies for germinal center (GC) B and plasmablast cells. Murine GCB cells were defined as B220$^+$CD4$^-$GL-7$^+$Fas$^+$, and plasmablast as B220$^+$CD4$^-$CD138$^+$.

Figure. S12

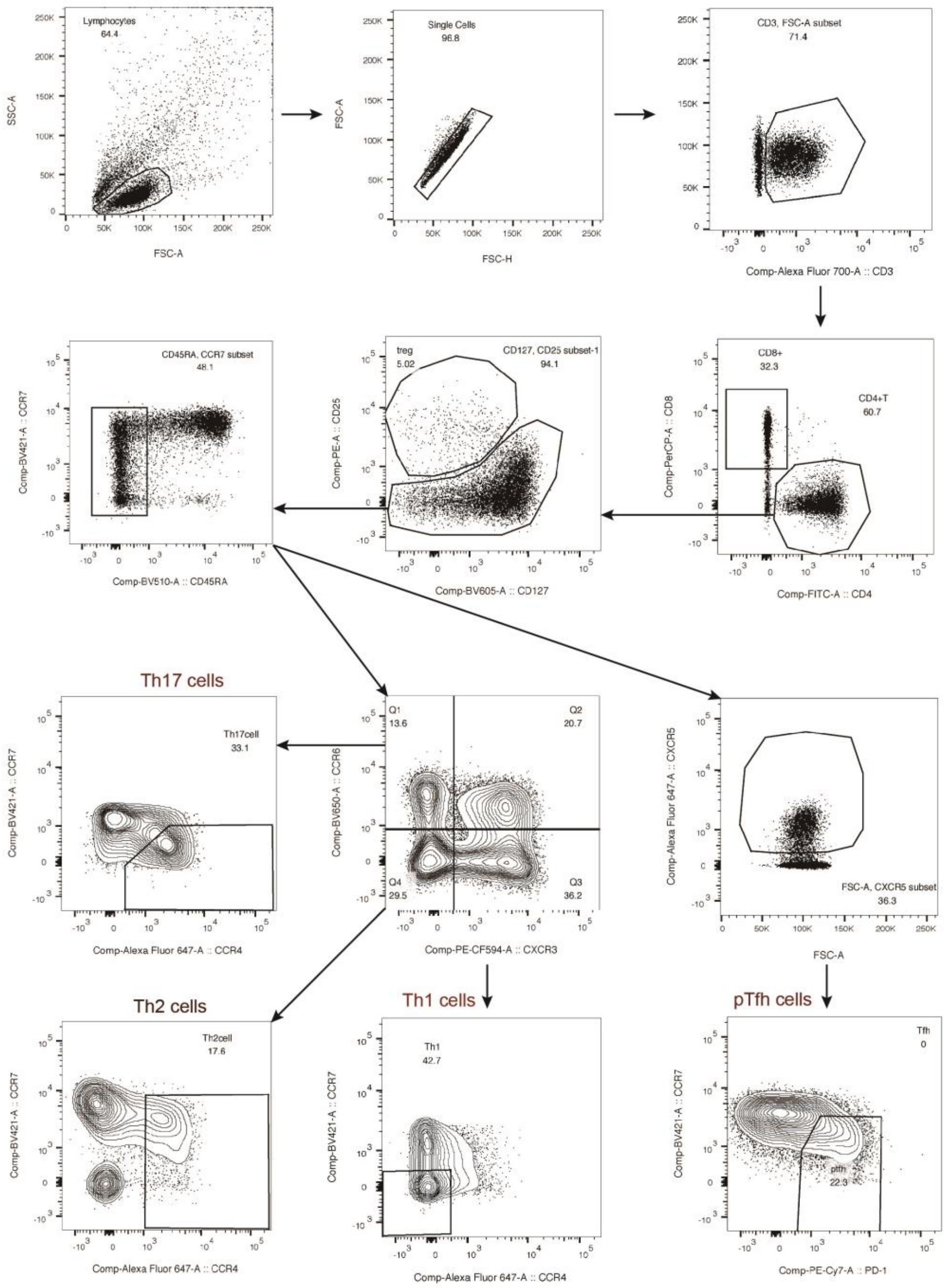

Figure S12. Schematic diagram displaying the gating strategies for T lymphocyte subsets in human. The human T immune cell subsets were identified as follows: Treg were marked as CD3+CD4+CD25^{hi}CD127^{low}, pTfh as CD3^{+}CD4^{+}CD25^{-}CD45RA^{-}CXCR5^{+}CCR7^{low}PD1^{+}, Th1 as CD3^{+}CD4^{+} CD25^{-}CD45RA^{-}CCR7^{low}CCR6^{-}CXCR3^{+}CCR4-, Th2 as CD3^{+}CD4^{+} CD25^{-} CD45RA^{-}CCR7^{low}CCR6^{-}CXCR3^{-}CCR4^{+}, Th17 as CD3^{+}CD4^{+}CD25^{-}CD45RA^{-}

CCR7^{low}CCR6^{+}CXCR3^{-}CCR4^{+},

Figure. S13

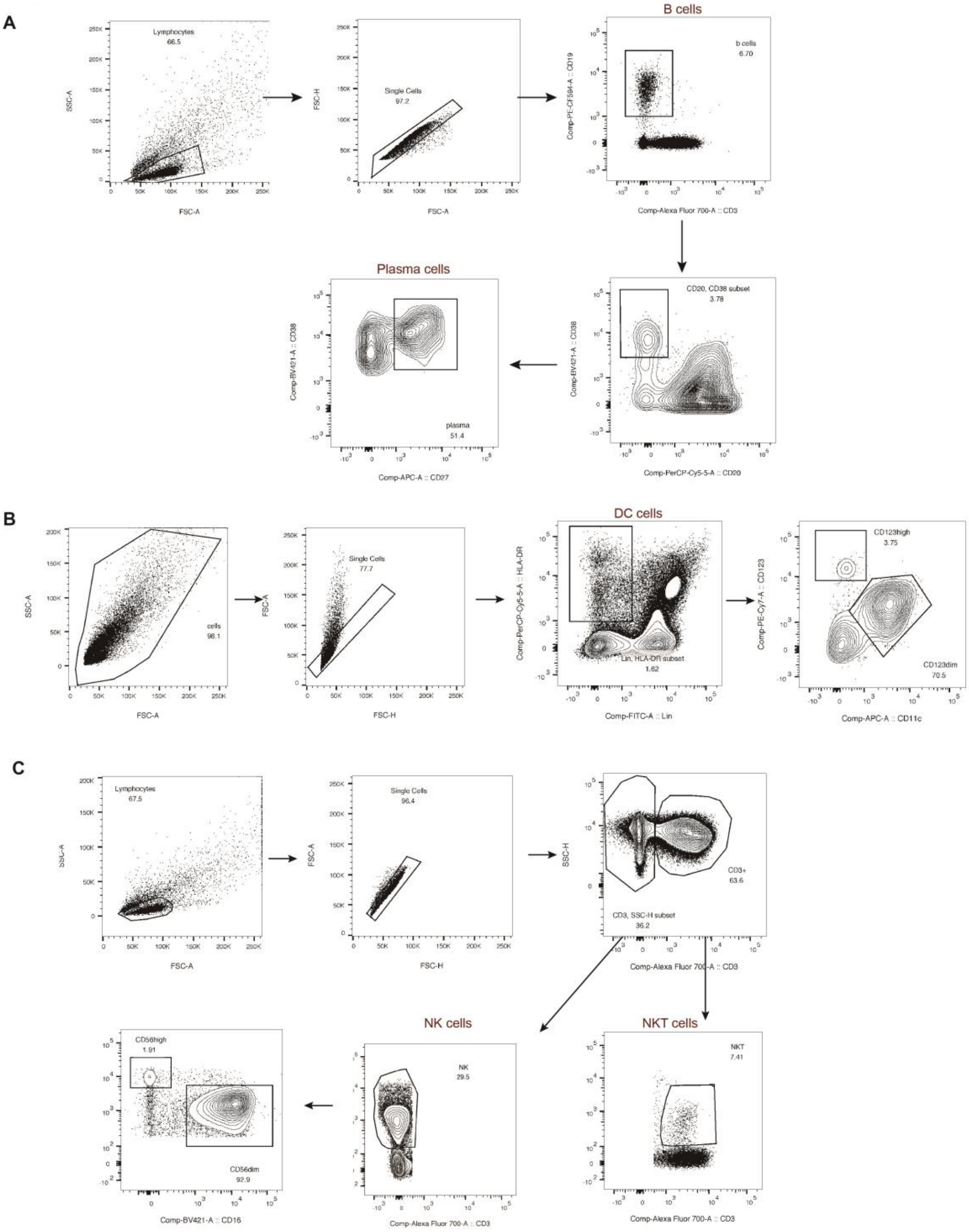

Figure S13. Schematic diagram displaying the gating strategies for immune cell subsets in human. Human plasma cells were identified as CD3^{-}CD19^{+}CD20^{-}CD38^{+}CD27^{+}, DC cells as Lineage-HLA-DR^{+}, CD123^{hi}DC cells as Lin-HLA-DR^{+}CD123^{hi}CD11C$^{-/dim}$, CD123^{dim}DC cells as Lineage-HLA-DR^{+}CD123^{dim}CD11C^{+}, CD56^{hi}NK cells as CD3^{-}CD56^{hi}CD16$^{-/dim}$,

$CD56^{dim}$NK cells as $CD3^{-}CD56^{dim}CD16^{+}$, NKT cells as $CD3^{+}CD56^{+}$.
